# A Co-Designed and Bilingual WhatsApp-Based Chatbot Intervention (PflegeBot) for Informal Caregivers in Germany: Single-Arm Nonrandomized Feasibility Study

**DOI:** 10.2196/95268

**Published:** 2026-11-11

**Authors:** Hande Gencer, Emily Mena, Theresa Freund, Sebastian Budde, Hajo Zeeb, Benjamin Schüz, Tilman Brand

**Affiliations:** 1 Faculty 11 Human and Health Sciences, University of Bremen, Bibliothekstrasse 9, Bremen, 28359, Germany, 49 042121856917; 2 Department of Prevention and Evaluation, Leibniz Institute for Prevention Research and Epidemiology - BIPS, Bremen, Germany; 3 Department of Epidemiology and Health Monitoring, Robert Koch Institute, Berlin, Germany; 4 Department of Health Care Research, Institute for Public Health and Nursing Research, Bremen, Germany; 5 Faculty 8 Social Sciences, University of Bremen, Bremen, Germany; 6 Department of Administration, Leibniz Institute for Prevention Research and Epidemiology - BIPS, Bremen, Germany; 7 Health Sciences Bremen, University of Bremen, Bremen, Germany; 8 Department of Prevention and Health Promotion, Institute for Public Health and Nursing Research, Bremen, Germany

**Keywords:** informal caregivers, mental health promotion, feasibility studies, mobile health, digital health interventions, chatbots, usability, participatory research

## Abstract

**Background:**

Informal caregivers play a central role in long-term care but experience elevated risks of stress, depressive symptoms, and caregiver burden, alongside barriers to accessing preventive support. Low-threshold digital public health interventions may offer scalable support; however, evidence on their feasibility, usability, and contextual fit remains limited, particularly for heterogeneous caregiver populations.

**Objective:**

This study describes the development and implementation of PflegeBot, an intersectionality-informed WhatsApp-based chatbot intervention, and evaluates its feasibility, usability, and acceptability among informal caregivers in Germany.

**Methods:**

A single-arm, nonrandomized feasibility study with pre-post assessments was conducted between December 2023 and March 2024. Informal caregivers aged 18 years and older were recruited via community-based, institutional, and online channels. PflegeBot was a co-designed, bilingual (German and Turkish) WhatsApp-based chatbot that delivered 84 scheduled messages over 12 weeks. Content included caregiving information, self-care, and cognitive behavioral therapy–informed self-help, plus optional frequently asked questions and question functions. Baseline and full follow-up assessments were administered online. Feasibility was assessed using indicators of recruitment, chatbot initiation and message delivery, opt-out, and provision of follow-up measures. Usability was assessed using the System Usability Scale (SUS), and acceptability was assessed using study-specific items. Exploratory outcomes included perceived stress (4-item Perceived Stress Scale [PSS-4]), caregiver burden (4-item Zarit Burden Interview [ZBI-4]), depressive symptoms (Center for Epidemiologic Studies Depression Scale [CES-D]), and loneliness (6-item De Jong Gierveld Loneliness Scale [DJGLS-6]). Analyses were descriptive; no hypothesis tests were conducted. Open-ended follow-up feedback was examined using a systematic inductive descriptive approach.

**Results:**

Of 102 participants included in the baseline analysis sample, 84 (82%) were women. According to chatbot log data, message delivery was initiated for 67 participants. Forty-nine participants provided at least one follow-up measure, and complete SUS data were available for 46 participants. The mean SUS score was 82.45 (SD 14.86, 95% CI 78.03‐86.86; range 47.5‐100), and 35 out of 46 (76%) participants scored at or above the benchmark of 68. Overall satisfaction with PflegeBot had a mean rating of 4.02 out of 5 (SD 0.98; n=46). Among participants responding to the relevant item, 1 out of 45 (2%) reported using the optional FAQ function. Mean changes were −0.57 (95% CI −1.24 to 0.10; n=35) for perceived stress, 0.81 (95% CI −0.02 to 1.63; n=36) for caregiver burden, −2.38 (95% CI −5.42 to 0.66; n=34) for depressive symptoms, and −0.39 (95% CI −0.90 to 0.12; n=36) for loneliness. Open-ended feedback highlighted needs for more flexible delivery, greater tailoring, and easier navigation.

**Conclusions:**

PflegeBot could be delivered through WhatsApp, and participants providing follow-up data reported high usability and generally favorable satisfaction. Controlled evaluations with objective engagement data and assessment of differential reach and outcomes are warranted.

## Introduction

Informal caregivers are central to the long-term care system in Germany, in which approximately 86% of people requiring long-term care are cared for at home [1]. In 2023, an estimated 9.8 million people provided informal care [2], a number expected to increase due to demographic aging [2,3]. Informal caregivers are typically family members or close social contacts who provide practical, emotional, and organizational support alongside employment, family responsibilities, and other obligations [1,4]. Compared with noncaregivers, they report higher levels of depressive symptoms and perceived stress [5-7]. Transitioning into informal caregiving is associated with loneliness [8]. Caregiver burden may persist over an uncertain and potentially prolonged period [9].

Gender inequalities in informal caregiving are well-documented. Approximately two-thirds of all informal caregivers in Germany are women [1,4], reflecting similar patterns across Europe [10]. Compared with caregiving men, caregiving women consistently report poorer well-being and higher levels of caregiver burden and perceived stress [6,11-13]. These inequalities reflect structural and normative conditions, including labor market inequalities and gendered expectations surrounding family care responsibilities, which shape exposure to caregiving demands and access to supportive resources [14-16].

An intersectional perspective further highlights that caregiving experiences and access to support are shaped by intersecting social positions and circumstances, including migration history, socioeconomic position, age, and caregiving context [17,18]. Nearly one-third of Germany’s population has a migration history [19]. People of Turkish origin represent the largest group among people with a migration history [20], but this population is itself diverse. Caregiving experiences and support needs may vary according to migration histories, language proficiency and preferences, socioeconomic resources, family constellations, and caregiving circumstances [21].

European studies indicate that informal caregivers with a migration history make less use of formal long-term care and support and rely more heavily on family-based care [22-26]. Reported barriers include socioeconomic constraints [27-29], limited familiarity with care systems and entitlements [25,26], help-seeking stigma [30], and insufficient linguistic and sociocultural adaptation of existing services [31]. These factors may exacerbate caregiving demands and time pressure, particularly when care responsibilities compete with paid work and caregivers’ own health [32], a pattern that may especially be pronounced among caregiving women with a migration history [33,34]. Although evidence remains limited, available studies suggest poorer mental health outcomes [35,36] and a higher burden among caregivers with a migration history [37].

Despite substantial health needs, informal caregivers make limited use of preventive and health-promotion interventions [38,39], with reported barriers including time constraints, informational gaps, and limited accessibility [39]. Access may further be restricted among caregivers experiencing intersecting disadvantages, including caregivers with a migration history [40].

Digital public health interventions (DPHIs) may improve access by providing low-threshold, flexible, and location-independent support [24,32,40-42]. Existing evidence suggests potential mental health benefits for informal caregivers [41,43], including for depressive and anxiety symptoms [44,45], perceived stress, and quality of life [46], although findings are mixed and feasibility evidence remains limited [47-50]. DPHIs include chatbot interventions, ranging from rule-based systems with predefined dialogue flows to AI-based agents that enable open-ended interaction [51,52]. Early studies have reported generally high user satisfaction and preliminary mental health benefits, although study quality varied [51,53,54]. Caregiver-focused examples include the educational chatbot integrated within CareHeroes [55], which was its most frequently used feature, and HIGEA [56], a conversational agent for assessing caregiver burden. However, DPHIs are not inherently beneficial. Differences in digital literacy and access, usability, language, and cultural appropriateness may limit uptake and reinforce existing inequalities [57,58]. Evidence on the usability, acceptability, and contextual fit of chatbot-based interventions for informal caregivers remains limited, particularly for bilingual, culturally adapted interventions designed for socially diverse informal caregivers [51,55].

Against this background, we developed PflegeBot, a bilingual WhatsApp-based chatbot intervention for informal caregivers in Germany. PflegeBot was developed as part of the M-Gender project (“Mental Health and Gender: Development and Testing of a Digital Intervention for Health Promotion among Informal Caregivers”) and co-designed with informal caregivers, researchers, and health and social care stakeholders. This study aimed to assess the feasibility, usability, and acceptability of PflegeBot among German- and Turkish-speaking informal caregivers. Changes in perceived stress, depressive symptoms, caregiver burden, and loneliness were examined as exploratory outcomes.

## Methods

### Study Design and Period

A single-arm, nonrandomized feasibility study with pre-post measurements was conducted to assess a chatbot intervention for informal caregivers. The study focused on feasibility, usability, and acceptability and was not designed to formally test intervention effectiveness [59]. Recruitment activities began in October 2023. Due to technical delays, participant-facing intervention delivery began in December 2023. After completing the baseline survey, participants entered the 12-week intervention on a rolling basis. Intervention delivery and follow-up data collection took place until the end of the study in March 2024.

### Reporting Guidance

The study was reported in accordance with the CONSORT-EHEALTH (Consolidated Standards of Reporting Trials of Electronic and Mobile Health Applications and Online Telehealth; V1.6.1) checklist, adapted for a nonrandomized, single-arm feasibility study [60], and the TIDieR (Template for Intervention Description and Replication) checklist [61]. The completed checklists are provided in Checklist 1 and Checklist 2.

### Study Setting

The intervention was developed and coordinated in Bremen, Germany, a socioeconomically and ethnically diverse city of approximately 700,000 inhabitants [62]. The feasibility study was open to informal caregivers residing across Germany; however, recruitment activities were concentrated in Bremen.

### Participants and Eligibility

Eligible participants were adults (≥18 years) who provided unpaid, nonprofessional care to another adult, had access to a smartphone with WhatsApp installed, had sufficient German or Turkish literacy, and were able to provide informed consent. Individuals caring exclusively for their own minor children were not eligible.

### Recruitment and Enrollment

Participants were recruited through local and regional organizations providing caregiving, migration-related, educational, self-help, health-promotion, and employment-related services. Recruitment partners included the Dementia Information and Coordination Center (Demenz Informations- und Koordinationsstelle), the Center for Migrant and Intercultural Studies (Zentrum für Migranten und Interkulturelle Studien), the Bremen Foundation of Nursing Homes and Residences, and the Bremen Adult Education Center, with additional support from the Bremen Chamber of Employees and trade unions.

Study information was disseminated through newsletters, mailing lists, social media, and in-person events. To enhance reach among caregivers of Turkish origin, community-based recruitment was conducted in settings such as mosques, cultural centers, and Turkish-speaking caregiver groups. Study materials were available in German and Turkish. Interested individuals accessed study information through a QR code or contacted the study team directly. At the end of the survey, participants received access information for the WhatsApp chatbot.

### Sample Size Considerations

A target sample size of 100 participants was defined. This target was chosen pragmatically based on the planned recruitment period and expected recruitment capacity and was considered sufficient for a descriptive assessment of feasibility, usability, and acceptability. No formal power calculation was conducted because the study was designed to assess feasibility, usability, and acceptability [59].

### Intervention Development

A needs assessment informed by qualitative interviews with 27 informal caregivers from diverse migration and educational backgrounds identified priorities related to caregiving information, psychosocial support, stress management, navigation of the long-term care system, and culturally sensitive services. Detailed methods and findings will be reported separately.

Intervention development was further guided by a co-design approach [63]. Three co-design workshops—2 conducted in German and 1 in Turkish—and additional stakeholder consultations were held between July 2022 and September 2023. Informal caregivers, stakeholders, and researchers jointly refined the proposed chatbot content and delivery formats. A review of relevant literature on digital and psychosocial caregiver interventions complemented the co-design process.

Development was guided by 4 principles: supporting caregivers’ mental well-being, using existing digital infrastructures, incorporating evidence-informed content, and providing culturally adapted content in German and Turkish. Content was developed collaboratively by informal caregivers, stakeholders, and an interdisciplinary research team with expertise in public health, psychology, social sciences, and digital media design. An intersectionality-informed perspective was applied to consider how gender, migration history, language, and socioeconomic circumstances may shape caregiving experiences and access to support [40].

### Intervention Description

#### Delivery and Format

PflegeBot was a 12-week WhatsApp-based chatbot delivered through the Chatfuel platform. Participants received 84 scheduled core messages, corresponding to one message per day, on their personal smartphones. During onboarding, participants selected German or Turkish, and all subsequent content was delivered in the selected language. Both versions provided equivalent content and functions and were culturally adapted for their respective target groups. A keyword-based FAQ function, accessed by typing the command “ask,” provided access to caregiving-related information. An optional question function—accessible via free-text input or a “help” command—allowed participants to submit free-text questions related to technical or informational issues, which were reviewed by the study team during office hours. Participants could revisit previous content and pause or discontinue messages at any time. No substantive changes were made to the intervention content during delivery.

#### Intervention Content

The intervention comprised 7 modules grouped into 3 thematic domains: normalization and orientation, skills and coping, and empowerment and connection. The modules addressed caregiving information and skills, self-care and stress management, caregivers’ rights and entitlements, and access to formal and informal support. Content was delivered through text, audio, video, and web links and included selected cognitive behavioral therapy–informed techniques [64]. The intervention provided low-intensity self-help but did not include individualized clinical assessment, diagnosis, psychotherapy, or other clinical treatment. Table 1 provides an overview of the modules; further details on the intervention content and structure are presented in Multimedia Appendix 1.

**Table 1.** Thematic domains, modules, and content of the 12-week PflegeBot intervention.

Domain and module	Content
Normalization and orientation	
Focus on Us – Caregivers Share Their Stories	Short digital storytelling videos featuring informal caregivers’ experiences with caregiving and formal support services, intended to promote identification, reduce isolation, and normalize help-seeking
Navigating Support Services	Short animations explaining relevant support services, structures, and access pathways within the German long-term care system
Skills and coping	
Managing Dementia and Everyday Care Tasks	Training videos addressing common caregiving challenges, including behavioral difficulties in dementia care
Me Time – Self-Care and Stress Management	Video and audio exercises addressing relaxation, mindfulness, body-based practices, and stress management
Me Time – Behavior Change Techniques	Cognitive behavioral therapy–informed exercises and reflective prompts addressing activity planning, coping with stress, and managing caregiving-related challenges
Empowerment and connection	
We Have Every Right! – Rights and Entitlements	Information on caregivers’ legal rights, financial benefits, application procedures, and available institutional support
Have You Heard of…? – Community-Based Support	Short video portraits of services, initiatives, and organizations relevant to informal caregivers

### Data Collection and Measures

#### Data Collection

Baseline and full follow-up assessments were administered online using LimeSurvey. The full online follow-up survey was delivered through the final chatbot message at the end of the individual intervention cycle, followed by up to 2 reminders at 2-week intervals through chatbot messages or telephone. Participants contacted by telephone were offered an abbreviated follow-up assessment covering usability, acceptability, and open-ended feedback; health outcomes were not assessed by telephone.

Baseline and full follow-up assessments were administered online using LimeSurvey. The full online follow-up survey was delivered through the final chatbot message at the end of the individual intervention cycle, followed by up to 2 reminders at 2-week intervals through chatbot messages or telephone. Participants contacted by telephone were offered an abbreviated follow-up assessment covering usability, acceptability, and open-ended feedback; health outcomes were not assessed by telephone.

German- and Turkish-language questionnaires, including the standardized health outcome measures, were translated from English and prepared by the research team specifically for this study. Previously validated German- and Turkish-language versions of these measures were not used.

Operational project logs on the Chatfuel interface and WhatsApp administration records documented chatbot initiation, initiation of scheduled message delivery, inactivity or nondelivery, and discontinuation through opt-out. Log and survey records were linked using study IDs. Message-level engagement metrics, including whether individual messages were opened, whether linked content was completed, or how much time participants spent engaging with the intervention, were not available. Content-level exposure and sustained engagement could therefore not be assessed.

#### Feasibility Outcomes

Feasibility indicators included recruitment, chatbot initiation, initiation of scheduled message delivery, inactivity or nondelivery, recorded opt-out, and provision of follow-up measures.

#### Usability Outcomes

Usability was assessed using the 10-item System Usability Scale (SUS) [65]. Items were rated on a 5-point Likert scale ranging from 1 (strongly disagree) to 5 (strongly agree) and scored according to the standard procedure, resulting in a total score ranging from 0 to 100, with higher scores indicating better usability. A total score was calculated only for participants with complete responses to all 10 items. A score of 68 or higher was used as a descriptive benchmark for acceptable usability.

#### Acceptability Outcomes

Acceptability was assessed using 6 study-specific items. Five items assessed overall satisfaction (“Overall, how much did you enjoy the PflegeBot?”), satisfaction with the daily messages (“How much did you enjoy the daily messages?”), appraisal of the frequently asked questions (FAQ) function (“How would you rate the question function with its information collection feature?”), support for future provision through health insurance providers (“How would you feel if your health insurance provider offered you a free PflegeBot in future?”), and likelihood of recommending PflegeBot (“How likely are you to recommend the PflegeBot to others?”). These items used 5-point response scales, with higher scores indicating more favorable ratings. One additional item asked whether participants had used the FAQ function (“Did you use the ‘question’ function to access the information collection?”), with the response options “Yes,” “No,” and “Did not know the function.”

#### Open-Ended Feedback

Optional open-ended feedback was collected through the online follow-up survey and during the abbreviated telephone follow-up (“Is there anything you’ve noticed whilst using the PflegeBot that you’d like to let us know about?”). The resulting material consisted of written survey responses and field notes documenting telephone responses. Supplementary formative consultation workshops conducted near the end of the feasibility phase are described in Multimedia Appendix 2.

#### Exploratory Health Outcomes

Health outcomes included depressive symptoms measured through the 20-item Center for Epidemiologic Studies Depression Scale (CES-D) [66], perceived stress measured through the 4-item Perceived Stress Scale (PSS-4) [67], caregiver burden measured through the 4-item Zarit Burden Interview (ZBI-4) [68], and loneliness measured through the 6-item De Jong Gierveld Loneliness Scale (DJGLS-6) [69]. The instruments, scoring procedures, and interpretation of scores are summarized in Table 2. Outcomes were assessed at baseline and follow-up. Participants were included separately in the paired analyses for each outcome when valid baseline and follow-up scores for that outcome were available.

**Table 2.** Exploratory health outcomes, instruments, scoring, and interpretation.

Instrument	Scoring	Interpretation
CES-D^a^	Items scored from 0 to 3, with positively worded items reverse scored; total score range: 0‐60	Higher scores indicate more depressive symptoms. Scores ≥16 were classified as indicating a higher likelihood of clinically relevant depressive symptoms [70].
PSS-4^b^	Items scored from 0 to 4, with positively worded items reverse scored; total score range: 0‐16	Higher scores indicate greater perceived stress. No cutoff was applied.
ZBI-4^c^	Items scored from 0 to 4; total score range: 0‐16	Higher scores indicate greater caregiver burden. Scores ≥8 were classified as high caregiver burden.
DJGLS-6^d^	Items with response options “yes,” “more or less,” and “no”; for negatively worded items, “yes” and “more or less” were scored 1, “no” was scored 0; for positively worded items, “yes” was scored 0, “more or less” and “no” were scored 1; total score range: 0‐6	Higher scores indicate greater loneliness. No cutoff was applied.

a CES-D: Center for Epidemiologic Studies Depression Scale.

b PSS-4: 4-item Perceived Stress Scale.

c ZBI-4: 4-item Zarit Burden Interview.

d DJGLS-6: 6-item De Jong Gierveld Loneliness Scale.

#### Sociodemographic and Caregiving Characteristics

Baseline sociodemographic characteristics included age, gender, country of birth, partnership and cohabitation status, employment status, highest educational attainment, and difficulty managing on their current household income. Caregiving characteristics included the relationship to the care recipient, the care recipient’s age and dementia status, living arrangements, receipt of professional care, the primary language used with the care recipient, duration of caregiving, and average weekly caregiving hours.

### Data Analysis

Substantially incomplete records, characterized by both missing age and gender information, were excluded. Records from respondents who did not meet the definition of informal adult caregiving were also excluded. Participant flow and availability of follow-up measures were summarized using the survey dataset. Chatbot initiation, initiation of scheduled message delivery, inactivity or nondelivery, and recorded opt-outs were summarized using operational project logs.

All quantitative analyses were descriptive. Continuous variables were summarized as means, SDs, and ranges, and categorical variables as counts and percentages. No missing values were imputed, and no between-group hypothesis tests were conducted.

SUS scores were summarized using the mean, SD, 95% CI, and range, together with the number and proportion of participants scoring at or above the descriptive benchmark of 68. Exploratory variation in SUS scores was described using subgroup-specific means and 95% CIs for categorical characteristics and Pearson correlation coefficients for continuous characteristics. Because some subgroups were small, the estimates were interpreted descriptively and not as evidence of group differences.

Study-specific acceptability items were analyzed using all available item-level responses and summarized using means, SDs, counts, and percentages as applicable. Item-specific denominators were reported because response availability differed across items.

Pre-post changes in health outcomes were calculated separately for each measure among participants with valid baseline and follow-up scores for that outcome. Baseline and follow-up means with corresponding standard deviations and within-participant mean changes with corresponding 95% CIs were reported. CIs were used to describe the precision of the estimates rather than to determine statistical significance. Quantitative analyses were conducted in Stata version 19.5 (StataCorp LLC). For methodological quality assurance, the authors used ChatGPT (GPT-5.6 Thinking; OpenAI) to audit, revise, and debug the Stata analysis code. Only privacy-sanitized code, the analytical specification, and aggregate execution logs were provided; the raw survey dataset was not uploaded. The authors reviewed and executed the final code locally in Stata 19.5 and retained full responsibility for the analyses. The prompts and audit documentation are provided in Multimedia Appendix 3.

Open-ended feedback was examined using a systematic inductive descriptive approach. One researcher fluent in German and Turkish reviewed all substantive responses in their original language, identified relevant statements, and developed preliminary category labels and definitions. Turkish-language responses were translated into German for secondary review. To enhance analytic trustworthiness, a second researcher reviewed all German-language responses, the German translations of Turkish-language responses, the category assignments, category definitions, and category summaries; the final classifications and descriptions were agreed upon through discussion. Researcher annotations and interpretations were excluded from the descriptive categorization. Paraphrased telephone field notes contributed to the categorization but were not used as illustrative quotations. The material was not independently double-coded. Further methodological details are provided in Multimedia Appendix 4.

### Ethical Considerations

The study was approved by the Ethics Committee of the Bremen Medical Association in July 2023 (application number 866). All participants received written study information and provided informed consent prior to participation. Participation was voluntary, and participants could withdraw at any time without consequences.

Survey data were collected through LimeSurvey, stored on secure institutional servers, and linked across assessment points using pseudonymized participant identifiers. Survey responses and health outcome data were not collected or stored through WhatsApp or Chatfuel. Participants were informed that Chatfuel and WhatsApp were used for intervention delivery, and data minimization principles were applied in accordance with the General Data Protection Regulation. Participation was free of charge, and no financial compensation or incentives were provided.

## Results

### Feasibility Outcomes: Participant Flow and Intervention Uptake

A total of 140 individuals entered the survey dataset. After exclusion of 30 (21%) substantially incomplete records, with age and gender information missing, and 8 (6%) additional records that did not meet the definition of informal adult caregiving, the baseline analysis sample comprised 102 participants.

According to project logs, 70 initiated the chatbot intervention after completing the baseline survey. Scheduled message delivery started for 67 participants; inactivity or nondelivery was documented for the remaining 3 participants. One participant opted out after message delivery had begun.

Of the 102 participants included in the baseline analysis sample, 49 provided at least one follow-up measure, including usability, acceptability, open-ended feedback, or a health outcome. Complete SUS data were available for 46 participants. Outcome-specific paired analyses included 34 to 36 participants with valid baseline and follow-up scores for the respective health outcomes. Participant flow is summarized in Figure 1.

**Figure 1.**
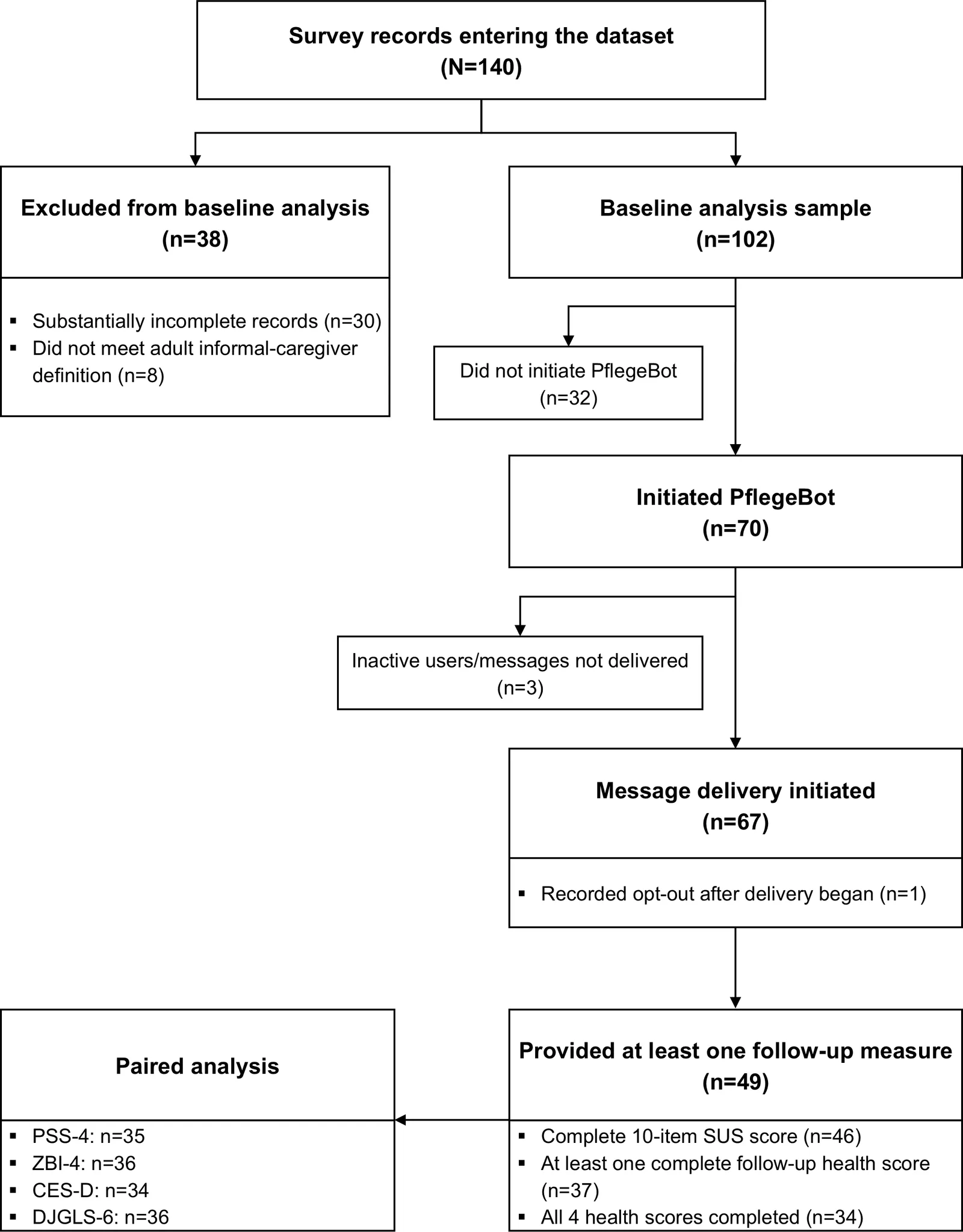
Participant flow chart through baseline analysis, PflegeBot initiation, message delivery, and follow-up assessment. CES-D: Center for Epidemiologic Studies Depression Scale; DJGLS-6: 6-item De Jong Gierveld Loneliness Scale; PSS-4: 4-item Perceived Stress Scale; SUS: System Usability Scale; ZBI-4: 4-item Zarit Burden Interview.

### Participant Characteristics

Table 3 presents the baseline characteristics of the baseline analysis sample and the subset of participants who provided at least one follow-up measure. Percentages in Table 3 were calculated using the column totals and rounded to whole numbers; consequently, they may not sum to 100. Missing values and the available sample were reported where applicable. Participants were predominantly women and were born in Germany and most cared for a parent or a spouse or partner. High caregiver burden and elevated depressive symptoms were common at baseline.

**Table 3.** Baseline characteristics of the baseline analysis sample and the subset providing at least 1 follow-up measure.

Characteristic	Baseline analysis sample (N=102)	Participants providing at least 1 follow-up measure (n=49)^a^
Age (y), mean (SD); range	56.17 (12.24); 23‐82	58.61 (11.54); 28‐81
Gender, n (%)		
Women	84 (82)	42 (86)
Men	18 (18)	7 (14)
Living with a partner or spouse, n (%)		
Yes	71 (70)	35 (71)
No	29 (28)	13 (27)
Other	1 (1)	0 (0)
Missing	1 (1)	1 (2)
Country of birth, n (%)		
Germany	81 (79)	41 (84)
Turkey	13 (13)	5 (10)
Other	7 (7)	2 (4)
Missing	1 (1)	1 (2)
Education (highest), n (%)		
No vocational qualification	9 (9)	4 (8)
Vocational qualification	51 (50)	22 (45)
University degree	40 (39)	22 (45)
Missing	2 (2)	1 (2)
Employment status, n (%)		
Full-time	35 (34)	15 (31)
Part-time	24 (24)	11 (22)
Retired	28 (27)	15 (31)
Not employed	6 (6)	4 (8)
Other	8 (8)	3 (6)
Missing	1 (1)	1 (2)
Difficulty managing on household income, n (%)		
Difficulty	13 (13)	5 (10)
No difficulty	88 (86)	43 (88)
Missing	1 (1)	1 (2)
Relationship to care recipient, n (%)		
Spouse or partner	36 (35)	18 (37)
Parent	42 (41)	19 (39)
Parent-in-law	7 (7)	2 (4)
Other family member	4 (4)	3 (6)
Nonfamily relationship	4 (4)	3 (6)
Other or unspecified	9 (9)	4 (8)
Age of care recipient (y), mean (SD); range	77.10 (11.50); 28‐97	79.00 (8.47); 59‐96
Care recipient has dementia or Alzheimer disease, n (%)		
Yes	40 (39)	27 (55)
No	61 (60)	22 (45)
Missing	1 (1)	0 (0)
Care recipient receives professional help, n (%)		
Yes	46 (45)	22 (45)
No	48 (47)	22 (45)
Missing	8 (8)	5 (10)
Care recipient living situation, n (%)		
Lives with the caregiver	45 (44)	22 (45)
Lives in own home	41 (40)	19 (39)
Lives in other arrangement	16 (16)	8 (16)
Weekly caregiving hours, mean (SD); range; n	47.10 (57.91); 1‐168; 98	49.14 (60.48); 2‐168; 48
Duration of caregiving, n (%)		
≤1 year	20 (20)	10 (20)
>1 to 2 years	16 (16)	9 (18)
>2 to <5 years	33 (32)	13 (27)
≥5 years	31 (30)	16 (33)
Missing	2 (2)	1 (2)
Language used with care recipient, n (%)		
German	82 (80)	41 (84)
Turkish	18 (18)	7 (14)
Other	2 (2)	1 (2)
Caregiver burden at baseline, n (%)		
ZBI-4^b^ score ≥8	65 (64)	34 (69)
ZBI-4 score <8	36 (35)	14 (29)
Missing^c^	1 (1)	1 (2)
Depressive symptoms at baseline, n (%)		
CES-D^d^ score ≥16	51 (50)	26 (53)
CES-D score <16	44 (43)	20 (41)
Missing	7 (7)	3 (6)

a The follow-up column is a subset of the baseline analysis sample with at least 1 follow-up measure; all characteristics were measured at baseline; no between-group comparisons were performed.

b ZBI-4: 4-item Zarit Burden Interview.

c Missing ZBI and CES-D values reflect incomplete baseline item responses and not absence of follow-up participation.

d CES-D: Center for Epidemiologic Studies Depression Scale.

### Usability and Acceptability Outcomes

#### Usability

Complete SUS data were available for 46 out of 102 participants in the baseline analysis sample. The mean SUS score was 82.45 (SD 14.86, 95% CI 78.03‐86.86; range 47.5‐100); 35 of 46 (76%) participants scored at or above the descriptive benchmark of 68.

#### Variation in Usability

Table 4 presents descriptive variation in SUS scores across participant and caregiving characteristics. Mean scores were higher among women than men and among participants with a university degree than among the other education groups, but several estimates were based on small samples and had wide CIs.

**Table 4.** Exploratory variation in System Usability Scale scores by participant and caregiving characteristics.

Characteristic	n	Mean SUS score (95% CI)^a^	Pearson *r*^b^
Age (y)	46	—^c^	–0.13
Gender			
Women	40	83.7 (79.0‐88.3)	—
Men	6	74.2 (57.7‐90.6)	—
Education (highest)			
No vocational qualification	3	74.2 (40.0-108.4)^d^	—
Vocational qualification	22	79.8 (73.4‐86.2)	—
University degree	20	88.4 (82.6‐94.2)	—
Missing	1	—	—
Language used with care recipient			
German	39	81.7 (76.9‐86.6)	—
Turkish	6	84.6 (69.2‐99.9)	—
Other	1	Not estimated^e^	—
Weekly caregiving hours	46	—	0.05
Duration of caregiving			
≤1 year	10	82.3 (69.6‐94.9)	—
>1 to 2 years	8	79.7 (67.3‐92.1)	—
>2 to <5 years	13	86.0 (77.9‐94.0)	—
≥5 years	15	81.0 (72.6‐89.4)	—
Care recipient has dementia or Alzheimer disease			
No	21	83.1 (77.3‐88.9)	—
Yes	25	81.9 (75.0‐88.8)	—
Care recipient receives professional help			
No	19	82.9 (75.5‐90.3)	—
Yes	22	81.0 (73.9‐88.1)	—
Missing	5	—	—

a Values are mean System Usability Scale (SUS) scores with 95% CIs.

b Pearson *r* denotes the Pearson correlation coefficient for continuous characteristics.

c Not applicable.

d The 95% CI extends beyond the theoretical SUS range because it is a conventional t-based interval based on 3 observations.

e The “Other” caregiving-language category was retained in the overall analysis. However, it included only 1 participant, so no mean or 95% CI was estimated.

#### Acceptability and Satisfaction

Overall satisfaction with PflegeBot had a mean rating of 4.02 (SD 0.98, n=46), and satisfaction with the daily messages had a mean rating of 3.76 (SD 1.14, n=46). Support for future provision through health insurance providers had a mean rating of 4.13 (SD 1.24, n=45), and the likelihood of recommending PflegeBot had a mean rating of 4.13 (SD 1.02, n=46). Among 45 participants responding to the FAQ item, 1 (2%) reported using the function, 36 (80%) reported not using it, and 8 (18%) reported that they did not know the function. The FAQ function received a mean rating of 3.16 (SD 0.90, n=31). However, 30 of the 31 participants providing a rating also reported that they had not used the function; the rating should therefore be interpreted as a general appraisal.

#### Open-Ended Feedback

Open-ended responses were available from 27 participants: 21 provided written survey responses, 5 provided feedback during a telephone-administered follow-up assessment, and 1 provided both written and telephone feedback. Of these, 25 participants provided substantive feedback and were included in the descriptive categorization. The descriptive categorization identified 4 nonmutually exclusive categories: relevance and tailoring (n=15), delivery intensity and timing (n=14), perceived benefit and acceptability (n=10), and format, usability, and navigation (n=8). Feedback commonly concerned the fit of the content with participants’ caregiving situations and the flexibility of message frequency and timing. Positive comments referred to practical information, learning, and self-care exercises, whereas concerns included content length, device-related usability, and difficulty retrieving specific materials (see Multimedia Appendix 4).

### Pre-Post Changes in Health Outcomes

Paired sample sizes varied by outcome (Table 5). Mean perceived stress, depressive symptoms, and loneliness were lower at follow-up, whereas mean caregiver burden was higher. All 95% CIs for the within-participant mean changes included zero, indicating inconclusive data regarding the direction of the changes. Given the feasibility design and limited sample sizes, the findings were interpreted descriptively and not as evidence of intervention effectiveness.

**Table 5.** Exploratory pre-post changes in health outcomes among participants with complete paired data.

Outcome	Paired n^a^	Baseline, mean (SD)	Follow-up, mean (SD)	Mean change (95% CI)^b^
PSS-4^c^	35	8.03 (2.97)	7.46 (2.73)	−0.57 (−1.24 to 0.10)
ZBI-4^d^	36	8.92 (3.13)	9.72 (3.09)	0.81 (−0.02 to 1.63)
CES-D^e^	34	19.76 (10.61)	17.38 (12.69)	−2.38 (−5.42 to 0.66)
DJGLS-6^f^	36	3.00 (2.11)	2.61 (2.06)	−0.39 (−0.90 to 0.12)

a Outcome-specific complete baseline and follow-up pairs.

b Calculated as follow-up minus baseline; negative values indicate lower scores at follow-up.

c PSS-4: 4-item Perceived Stress Scale.

d ZBI-4: 4-item Zarit Burden Interview.

e CES-D: Center for Epidemiologic Studies Depression Scale.

f DJGLS-6: 6-item De Jong Gierveld Loneliness Scale.

## Discussion

### Principal Findings

This study assessed the feasibility, usability, and acceptability of PflegeBot, a bilingual, rule-based WhatsApp chatbot for informal caregivers in Germany. Regarding feasibility, the baseline recruitment target was reached, although the number of participants decreased across chatbot initiation, message delivery, and follow-up, indicating challenges in intervention uptake and follow-up participation. Among participants providing follow-up data, usability was high, and acceptability was rated favorably, although satisfaction with the daily messages was lower than the other acceptability ratings. Reported use of the FAQ function was limited.

Pre-post analyses indicated lower mean scores for perceived stress, depressive symptoms, and loneliness at follow-up and a higher mean caregiver-burden score. However, all CIs for the within-participant changes included zero. Given the single-arm design, loss to follow-up, limited paired samples, and heterogeneity in caregiving circumstances, these findings should not be interpreted as evidence of effectiveness.

Open-ended feedback indicated that intervention fit depended on the correspondence between delivery, content, and participants’ caregiving circumstances.

### Comparison With Prior Work and Interpretation

The favorable usability and acceptability ratings are broadly consistent with previous evaluations of digital interventions for informal caregivers [45,49]. At the same time, limited follow-up participation and the low reported use of the FAQ function point to challenges in sustaining participation and making interactive features sufficiently visible [71].

Participants differed in how well standardized daily delivery fitted their available time and current information needs. Some valued regular messages as reminders, whereas others reported information overload, insufficient time, or limited relevance to their immediate caregiving situation. Similar variation has been reported for other digital caregiver interventions [39,72]. Messenger-based interventions may therefore benefit from adjustable delivery and modular pathways that preserve the prompting function of regular messages while allowing participants to select content relevant to their circumstances [41,46,49,73].

Exploratory variation in SUS scores was based on small subgroups and imprecise estimates and therefore does not support conclusions about differences by education, gender, or caregiving characteristics. Nevertheless, evidence suggests that education, digital literacy, and familiarity with digital services may influence perceived usability [71]. These factors should remain a consideration for future interventions.

Mean caregiver burden increased slightly, whereas the other exploratory outcome means decreased; a similar pattern was reported by Ghani et al [48]. These estimates were imprecise and may reflect changes in caregiving circumstances or measurement variability or an increased awareness and recognition of caregivers’ own burden fostered by participation in the intervention. However, these mechanisms were not assessed, and given the study’s single-arm, nonrandomized design, no beneficial or adverse intervention effect can be inferred.

PflegeBot was developed through a participatory process intended to incorporate caregivers’ experiences and language-related needs into its content and delivery—an approach that has already proven promising in other DPHIs [63,71,73]. The study did not establish equitable accessibility or acceptability across gender, intervention language, or social groups. Nevertheless, the findings indicate that language, digital experience, education, available time, and caregiving circumstances should be considered jointly rather than as isolated determinants [18,29,74].

### Strengths and Limitations

Strengths of this study include the co-designed bilingual intervention, delivery through an established messaging platform, and evaluation under real-world conditions. Combining quantitative measures with open-ended follow-up feedback provided complementary descriptive information on acceptability, contextual fit, and intervention refinement, addressing gaps frequently identified in evaluations of digital caregiver interventions [41,49].

This study has several limitations. First, the single-arm feasibility design without a control group precludes conclusions about effectiveness. Second, the follow-up and paired-analysis samples were substantially smaller than the baseline sample. Because participants entered the intervention on a rolling basis, not all baseline participants had sufficient time to complete the full 12-week intervention and follow-up assessment before the study ended in March 2024. The reduction in sample size between baseline and follow-up therefore cannot be attributed solely to attrition, although selective follow-up participation cannot be excluded. In addition, more than one-fifth of the survey records were substantially incomplete and excluded from the baseline analysis. These records could not be adequately characterized or compared with the included sample, creating a further potential source of selection bias. Third, objective message- and content-level usage metrics were unavailable. Exposure to individual intervention components, content completion, and sustained engagement could therefore not be assessed. Fourth, the sample predominantly consisted of women, and the number of participants using the Turkish-language intervention was small, preventing reliable subgroup or intersectional analysis. Fifth, participants were recruited through diverse channels and self-selected into the study, while eligibility required access to a smartphone with WhatsApp. The sample may therefore have had greater digital familiarity, motivation, or readiness to use digital support than the broader population of informal caregivers. Sixth, the standardized health measures were administered using study-specific translations rather than previously validated German- and Turkish-language versions, and their semantic and psychometric equivalence was not formally evaluated. Finally, although the study was open to informal caregivers throughout Germany, recruitment activities were concentrated in Bremen, which may limit transferability to other settings.

### Implications for Future Research and Practice

The findings indicate that messenger-based chatbot interventions may complement existing caregiver support services when delivery and content can be adapted to caregivers’ changing circumstances and time constraints. Future development should therefore prioritize user control over intervention delivery and content selection and reduce barriers to navigation and interaction.

Future studies should use adequately powered, controlled designs and integrate objective engagement metrics alongside self-report measures. Mixed methods approaches remain important for contextualizing quantitative outcomes and for understanding engagement and nonengagement [47,71]. Evaluations should also examine differential reach, usability, follow-up participation, and outcomes across relevant social and caregiving contexts. In studies using rolling enrollment, the opportunity to complete the intervention and follow-up should be considered when defining follow-up denominators and reporting attrition.

Sustainable implementation of chatbot-based caregiver interventions will require clear organizational responsibility for content review, technical maintenance, user support, and data protection, particularly in light of ongoing challenges associated with implementing reimbursable digital care applications in Germany [75]. Dependence on commercial messaging platforms should also be considered an implementation risk requiring ongoing technical and organizational adaptation.

### Conclusions

This feasibility study indicates that PflegeBot could be delivered to informal caregivers through WhatsApp, although challenges were observed across intervention uptake and follow-up participation. Among participants providing follow-up data, usability was high and acceptability was generally favorable. Further development should focus on adaptable delivery, tailoring, and accessible interaction. Controlled studies are needed to assess effectiveness, sustained engagement, and differential reach across social and caregiving contexts.

## Supplementary material

10.2196/95268 Multimedia Appendix 1 Overview of the PflegeBot intervention content and modules.

10.2196/95268 Multimedia Appendix 2 Methods and supplementary findings for the participatory consultation workshops.

10.2196/95268 Multimedia Appendix 3 Generative AI–assisted Stata code audit: prompts, audit transcript, and validation documentation.

10.2196/95268 Multimedia Appendix 4 Methods and supplementary findings for open-ended follow-up feedback.

10.2196/95268 Checklist 1 CONSORT-EHEALTH checklist (V 1.6.1).

10.2196/95268 Checklist 2 TIDieR checklist.

## Abbreviations

CES-D: Center for Epidemiologic Studies Depression Scale
CONSORT-EHEALTH: Consolidated Standards of Reporting Trials of Electronic and Mobile Health Applications and Online Telehealth
DJGLS-6: 6-item De Jong Gierveld Loneliness Scale
DPHI: digital public health intervention
FAQ: frequently asked questions
PSS-4: 4-item Perceived Stress Scale
SUS: System Usability Scale
TIDieR: Template for Intervention Description and Replication
ZBI-4: 4-item Zarit Burden Interview
