# Supplementary material for: A Co-Designed and Bilingual WhatsApp-Based Chatbot Intervention (PflegeBot) for Informal Caregivers in Germany: Single-Arm Nonrandomized Feasibility Study
Source: JMIR Form Res. 2026 Nov 11;10:e95268. doi: 10.2196/95268 (PMC13754261; doi:10.2196/95268)
Supplement: Supplementary file 1 [file formative-v10-e95268-s001.pdf]

Hande Gencer, Emily Mena, Theresa Freund, Sebastian Budde, Hajo Zeeb, Benjamin Schüz, Tilman Brand

Overview of the PflegeBot intervention content and modules

Module 1: Focus on Us – Caregivers Share Their Stories

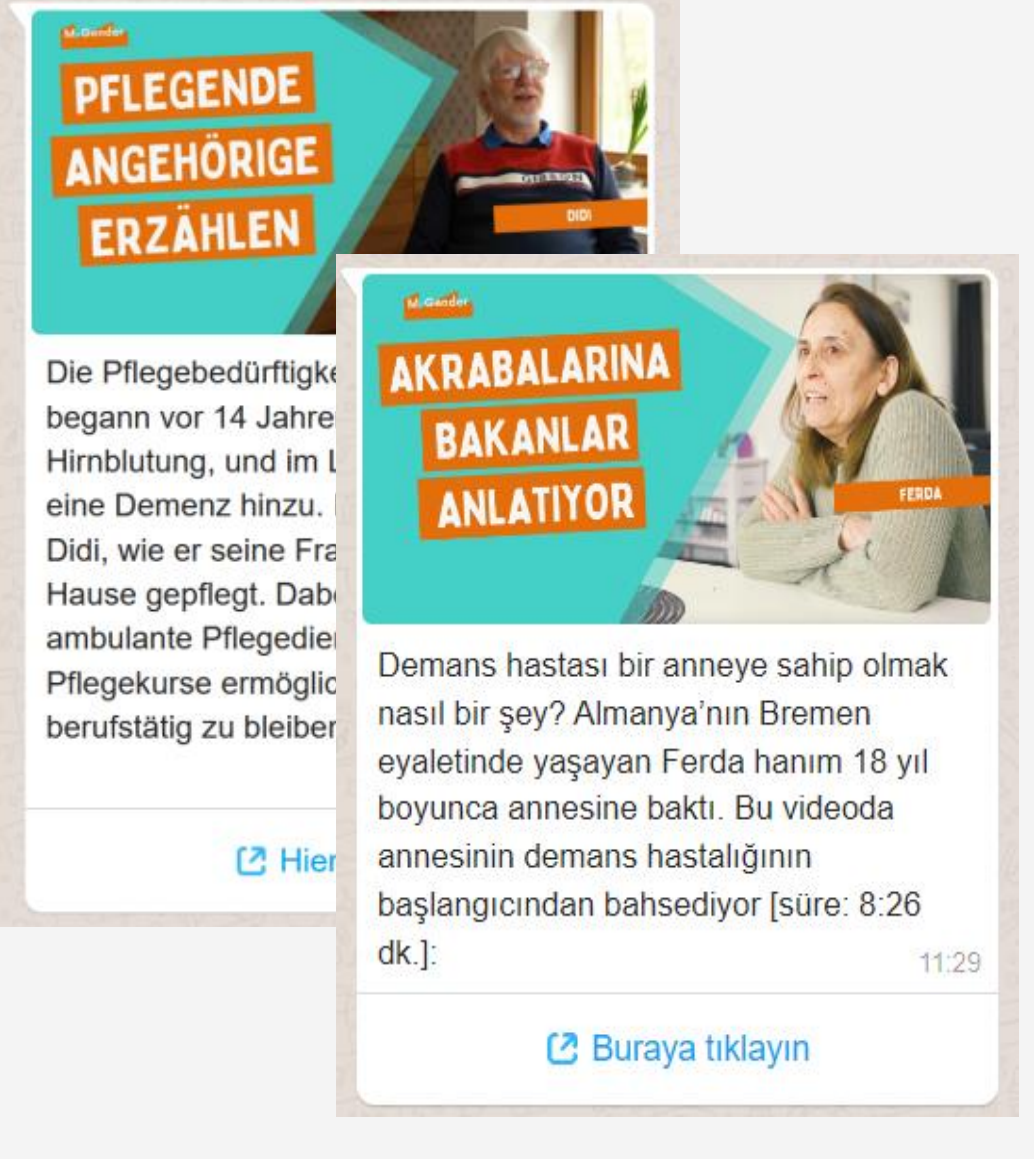

- Six self-produced videos with two German-speaking informal caregivers (one man, one woman, >60 years)
- Digital storytelling: narratives from the everyday lives of informal caregivers
- Every two weeks on Monday
- Turkish version: equivalent number of videos featuring a Turkish-speaking woman caregiver

Module 2: Navigating Support Services (“What is/are… again?”)

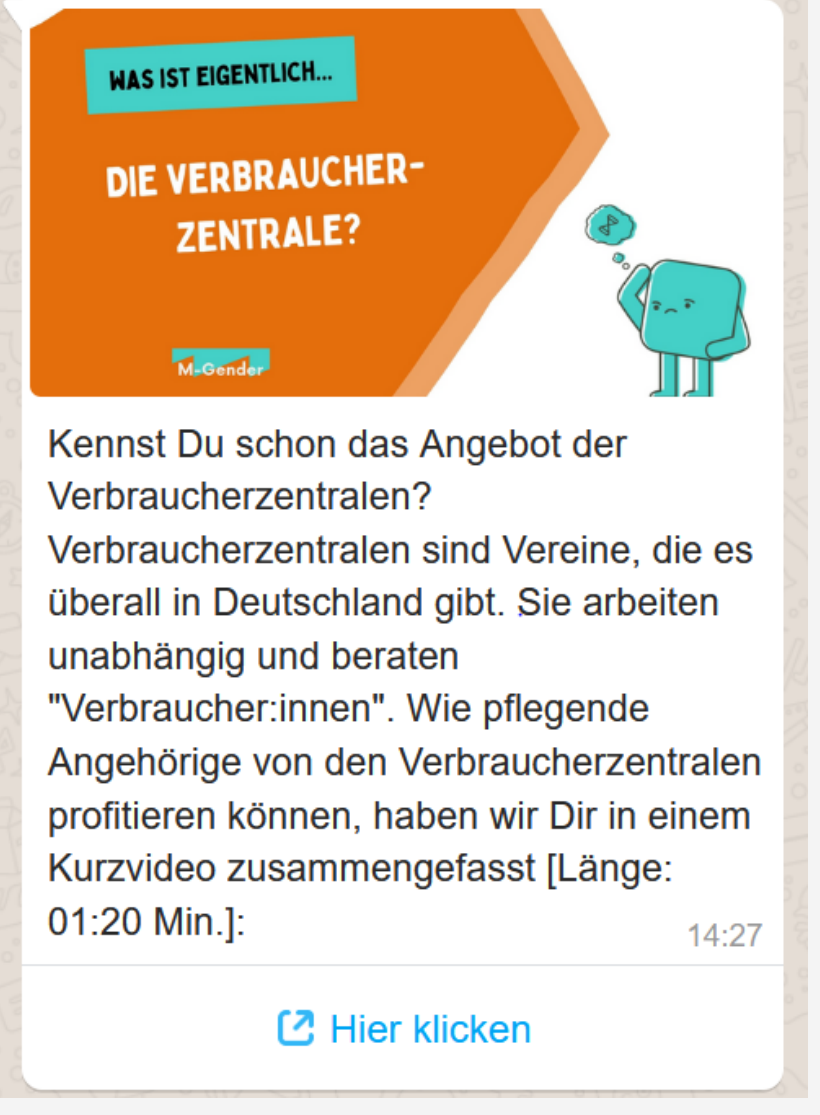

- Six self-produced animations on six different caregiver services at national and local level (Bremen)
  - Independent patient information offices
  - German Consumer Organizations
  - Care-sharing flat shares
  - Support groups for informal caregivers
  - Neighborhood Assistance
  - Care Support Centers
- Provide information on mostly free and low-threshold caregiver services
- Every two weeks on Monday
- Turkish version: translated animations

Module 3: Managing Dementia and Everyday Care Tasks

UCLA Health Caregiver Training [1]

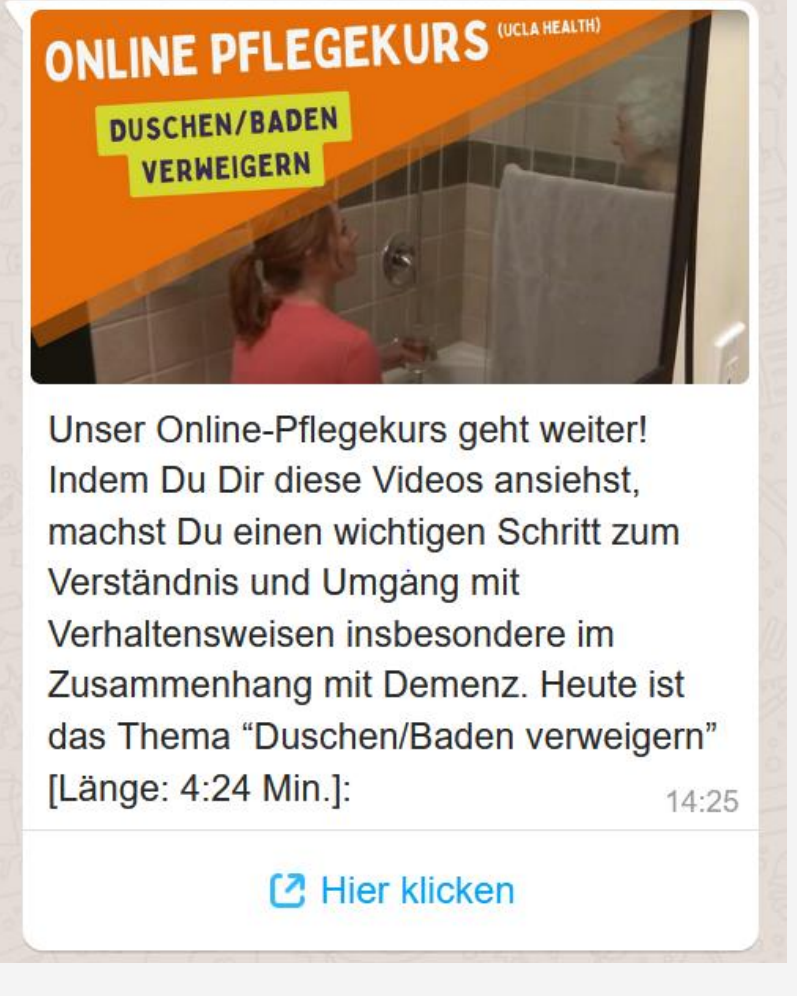

- 18 videos (8 UCLA, 19 PfiFf)
- Tuesdays
- Extras: other caregiver support and socio-culturally sensitive services
- Turkish versions: dubbed in Turkish (UCLA) and subtitled (PFifF)

PfiFf - Pflege zu Hause [2]

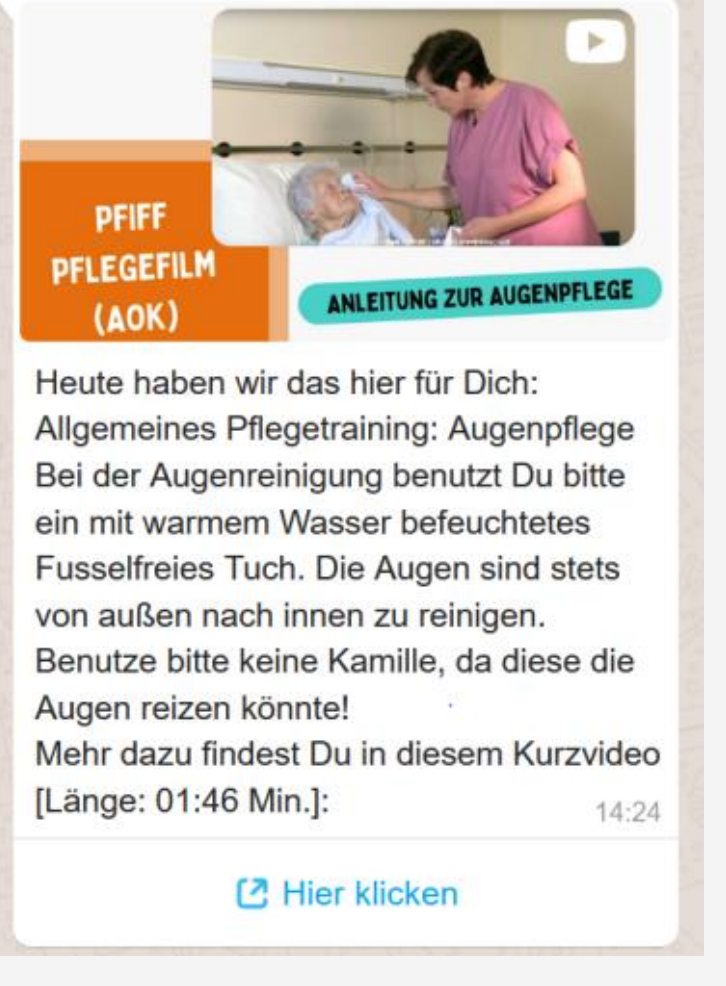

Modul 4: Me Time – Self-Care and Stress Reduction

Meditation/ Mindfulness

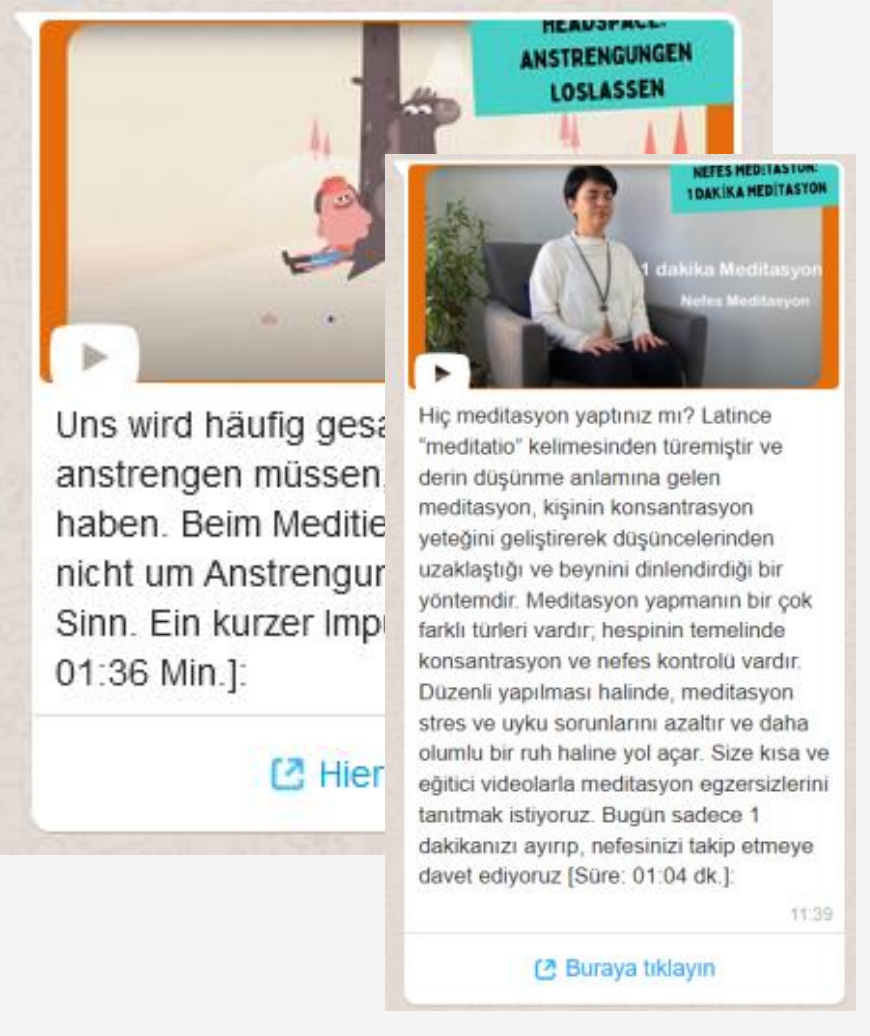

- 16 YouTube videos on meditation & relaxation
- 11 audio files on mindfulness exercises (or links to them)
- 10 online yoga courses from *Techniker* Health Insurance Fund [3]
- Wednesdays and Saturdays, usually two videos per post
- Turkish version: 34 Turkish YouTube videos on meditation, mindfulness, and progressive muscle relaxation

Movement

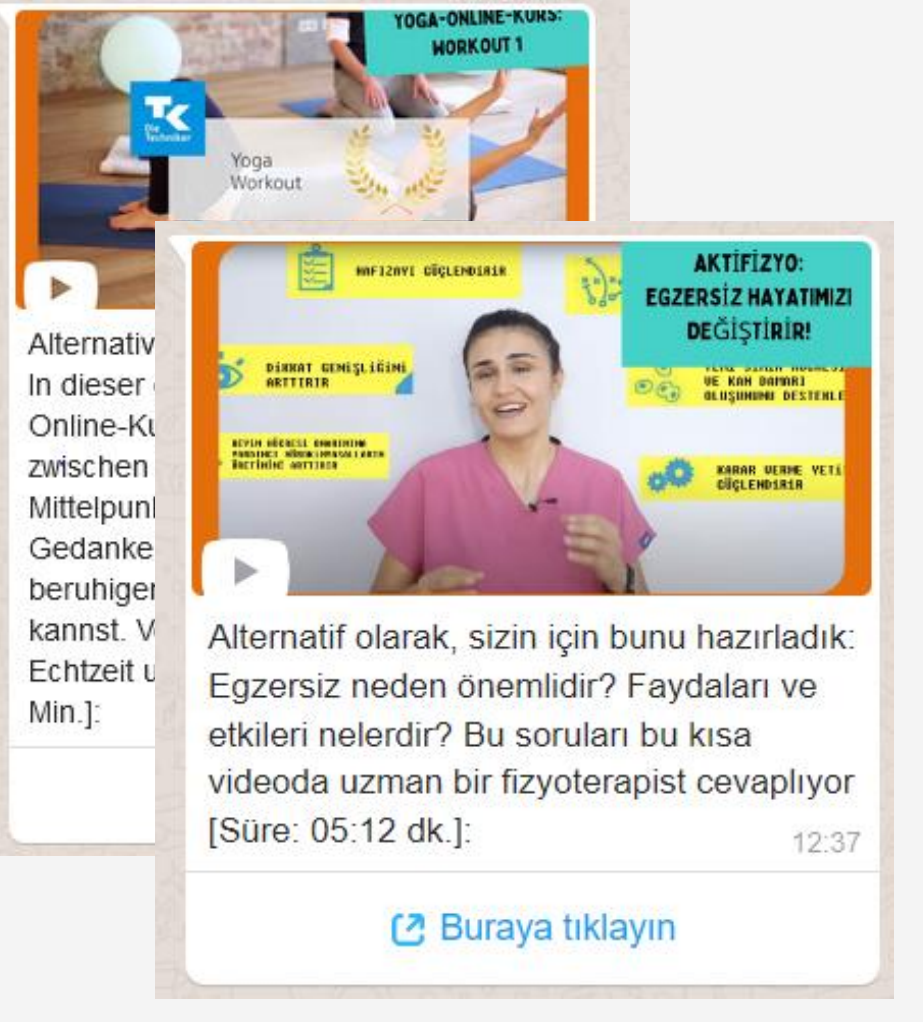

Module 5: Me Time – Behavior Change Techniques

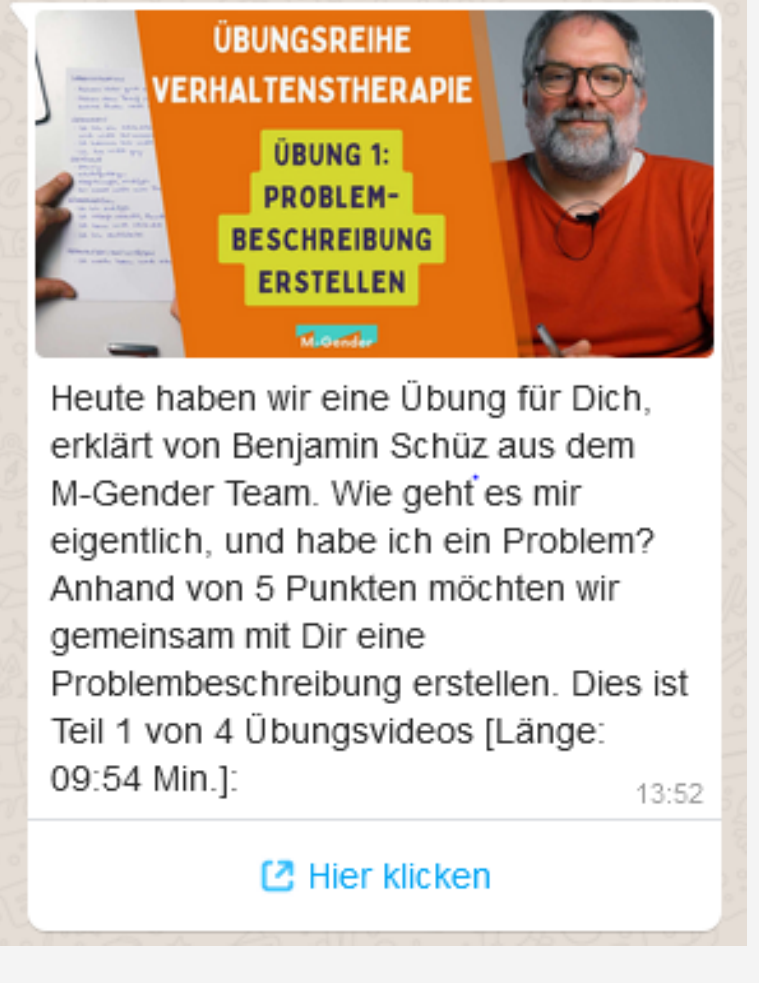

- Four self-produced videos with a mental health expert
- Behavioral therapy exercise series: (1) Describe the problem, (2) Create an activity plan, (3) Change activities, (4) Start making changes
- Sundays
- Turkish version: four self-produced animations, with Turkish voiceovers

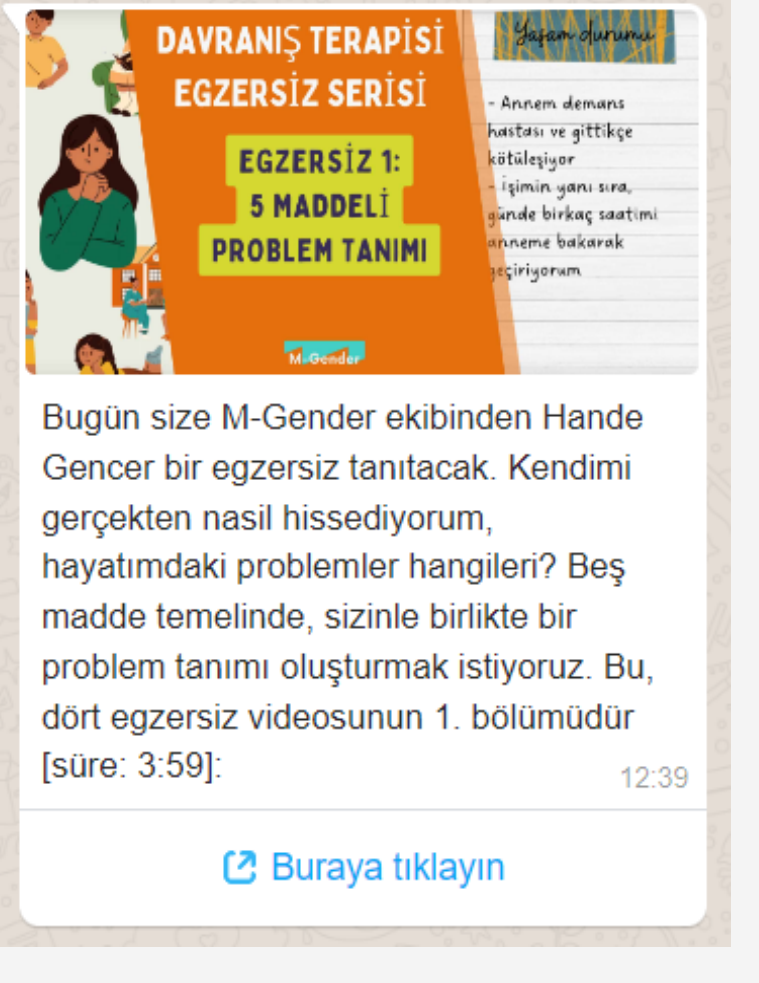

Module 6: That Is Our Right! – Rights and Entitlements

Sozialverband VdK [4]

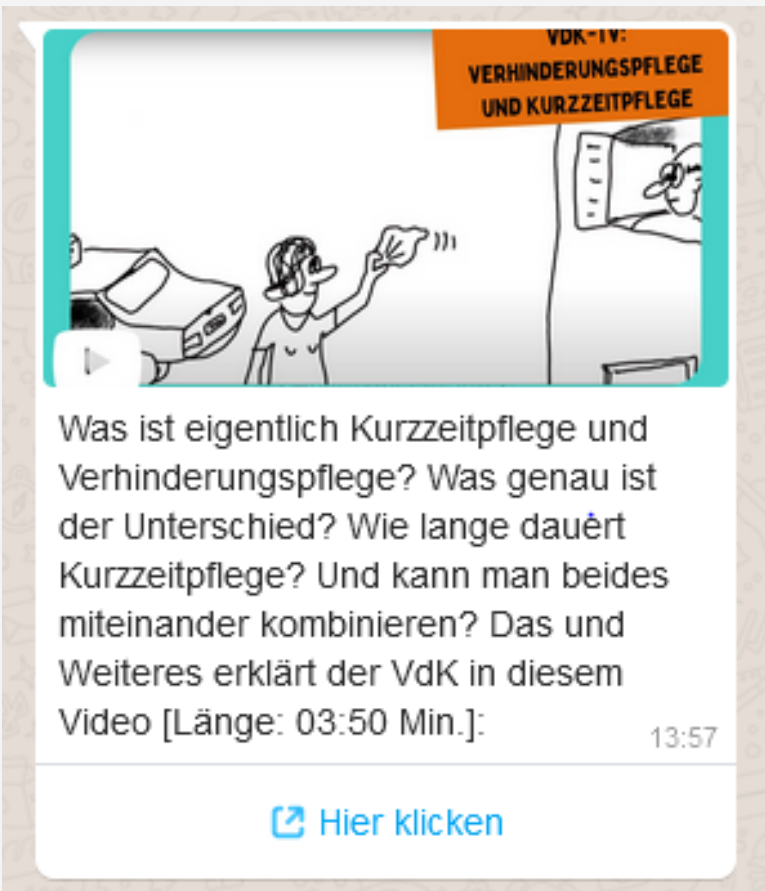

- 13 videos (one additional offer from the German Alzheimer's Association) from the Social Association VdK Germany on the rights of family caregivers
- Thursdays
- Turkish versions: dubbed in Turkish and subtitled

Andere Quellen

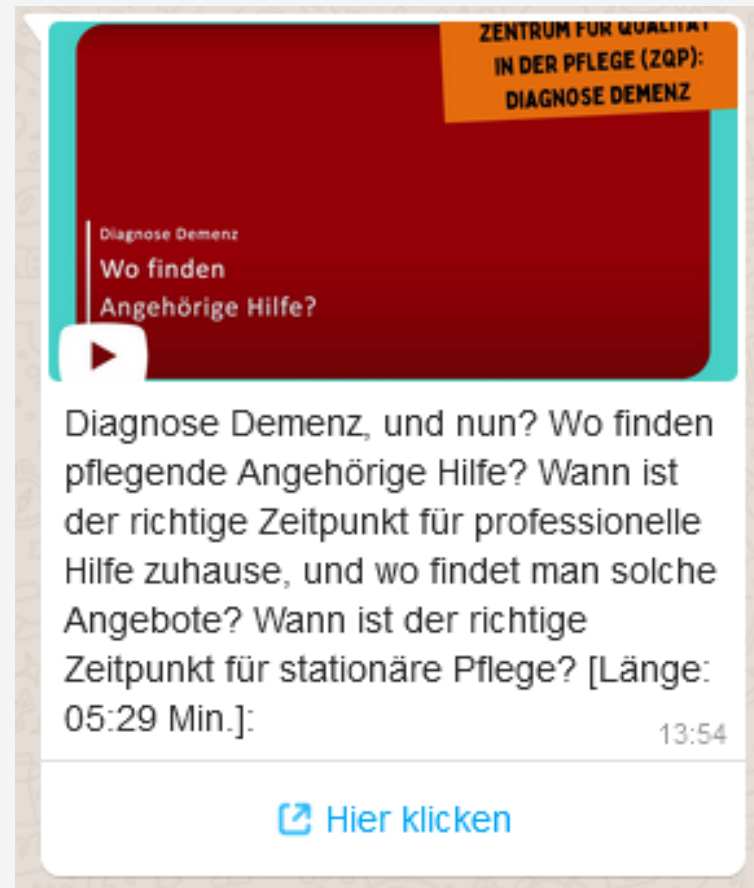

Module 7: Have You Heard of…? – Community-Based Support

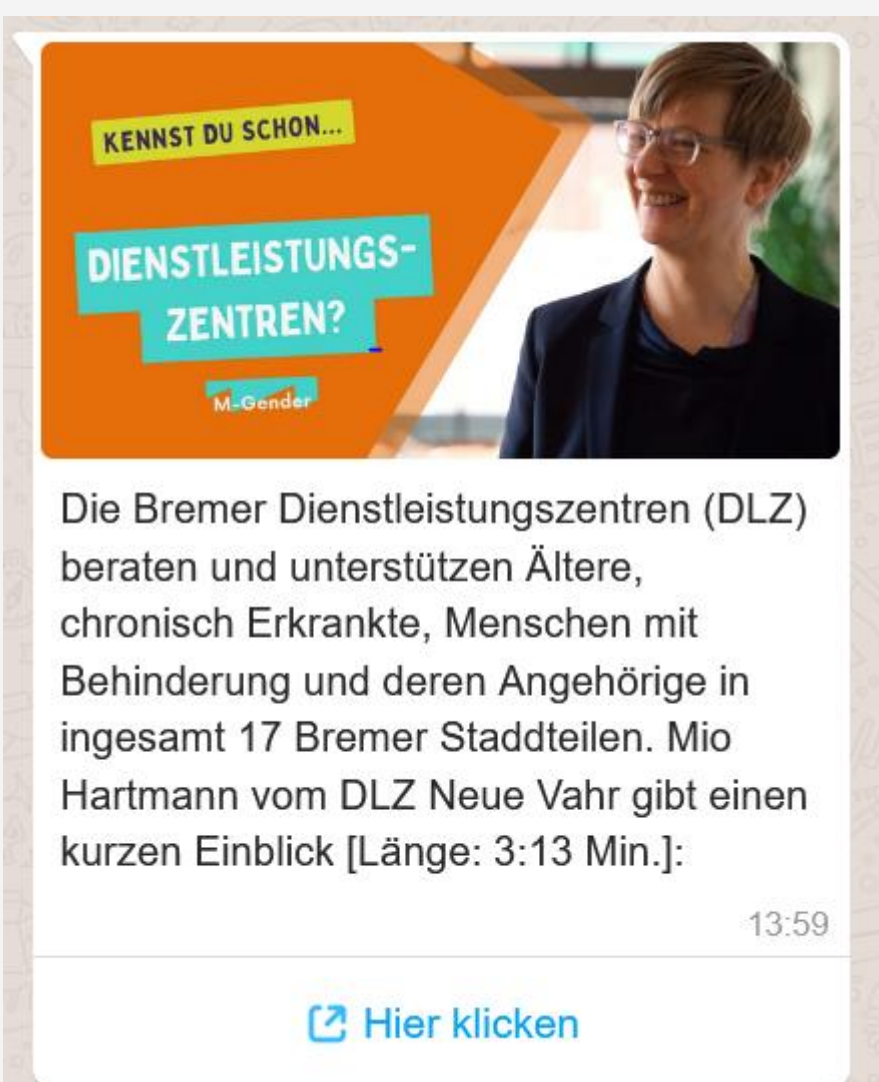

- 12 self-produced videos introducing selected institutions in Bremen/ Germany
  - Neue Vahr Service Center
  - Bremen Chamber of Employees
  - Bremen Care Support Centers
  - Center for Migrants and Intercultural Studies (ZIS)
  - Social Association VdK
  - Bremen Family Network
  - Dementia Information & Advice Center (DIKS)
  - *Frauengesundheit in Tenever*
  - Bremen Adult Education Center
  - Help Line (DIKS) Bremen
  - Health mediators of the State Association for Health Lower Saxony
  - *Stiftungsdorf Hemelingen*
- Fridays
- Turkish versions: Turkish-speaking experts, if available, and subtitles for the rest

Miscellaneous – German version

Documentaries

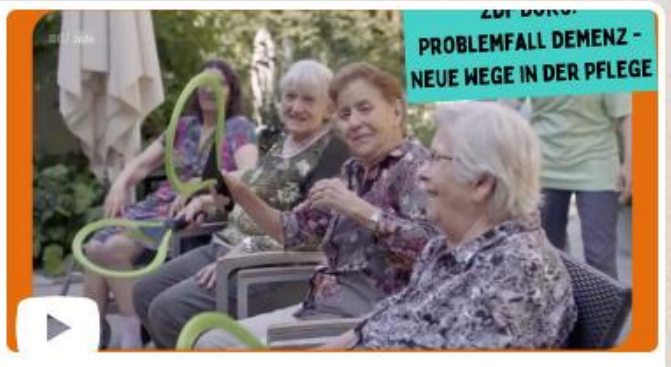

Heute möchten wir Dir eine Dokumentation von ZDFinfo mit dem Titel "Problemfall Demenz - Neue Wege in der Pflege" empfehlen: "Die Pflege älterer Menschen mit Demenz ist ein wachsendes gesellschaftliches Problem. Wie kann den Betroffenen - Erkrankten wie Angehörigen - ein erfülltes Leben ermöglicht werden?" [Länge: 43:41 Min.]: 14:06

Hier klicken

Websites

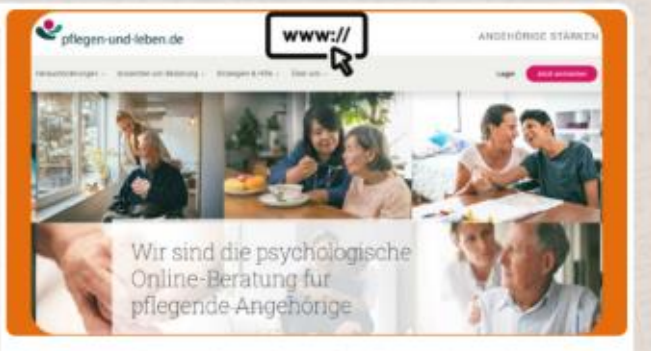

Kennst Du schon das Online-Angebot von Pflegen und Leben? Es handelt sich um ein psychologisch gestütztes Portal mit nützliche Informationen zur Entlastung und Selbstpflege, sowie Hilfestellungen für den Umgang mit schwierigen Situationen in der Pflege. Hier kannst Du Dich schriftlich mit Expert:innen austauschen. Manchmal hilft es, sich Sorgen und Probleme von der Seele zu schreiben, probiere es doch mal aus: 14:09

Hier klicken

Books and other

Heute haben wir einen Lesetipp für Dich: Inge Jens hat in ihrem Buch "Langsames Entschwinden - Vom Leben mit einem Demenzerkrankten" Briefe an Freund:innen und Bekannte dokumentiert, in denen sie emotional von der Demenzerkrankung ihres Mannes, Walter Jens, berichtet: "Einfühlsam und respektvoll beschreibt Inge Jens die Veränderungen, die durch die fortschreitende Krankheit verursacht wurden; und offen reflektiert sie die eigene Unsicherheit, wie man sich dem Kranken gegenüber verhalten soll." 14:05

Hier klicken

Miscellaneous – Turkish version

Documentaries

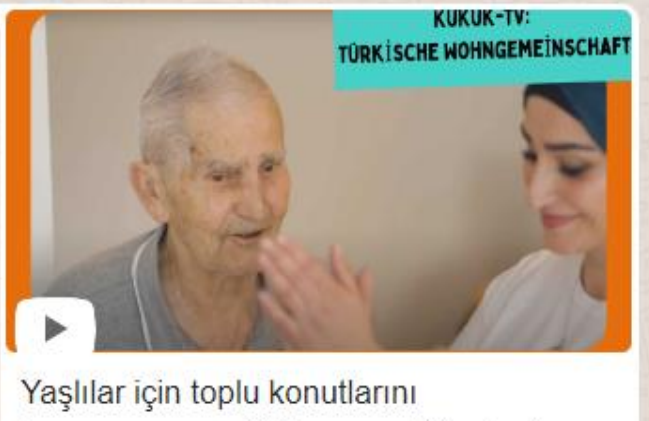

Yaşlılar için toplu konutlarını tanıyormusunuz? Almanca, "Senior:innen-Wohngemeinschaft/WG" veya "Pflege-WG" olarak tanılmaktadır. Burada yaşlılar tanıdık bir çevrede bağımsız olarak yaşayabilirler. Bu videoda Stuttgart'taki "Emin Eller" projesi tanıtılmaktadır [Süre: 10:00 dk]: 12:56

Buraya tıklayın

Websites

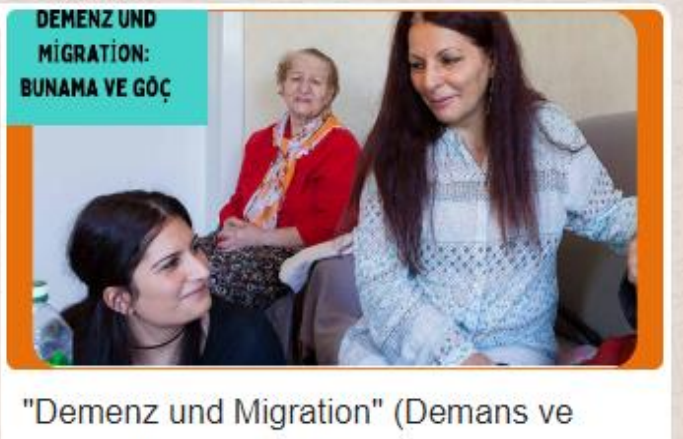

"Demenz und Migration" (Demans ve Göç) adlı internet sitesini tanıyormusunuz? Alman Alzheimer Derneği tarafından Almanya'da demans, bakım ve göç konularında derlenen kapsamlı bilgiler yer alıyor. İncelemenizi tavsiye ederiz: 12:57

Buraya tıklayın

Books and other

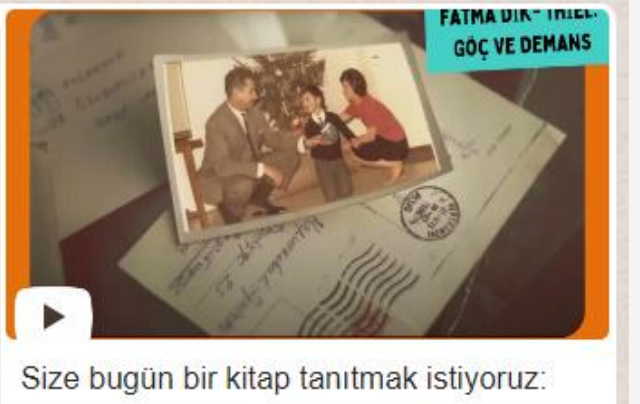

Size bugün bir kitap tanıtmak istiyoruz: "Göç ve Demans - Maziye Bir Bakıver" adlı kitapta yazar Fatma Dik-Thiel, Almanya'ya göç eden ve Almanyada yaşlanan ilk kuşağın göç hikayelerini, gurbet-sıla arasında yaşanan hayatlarını, Demansla mücadelerini ve yaşlıların huzurevindeki yaşam öykülerini anlatıyor. [Süre: 04:24 Dk.] 12:58

Buraya tıklayın

“Ask” free-text & FAQ functions

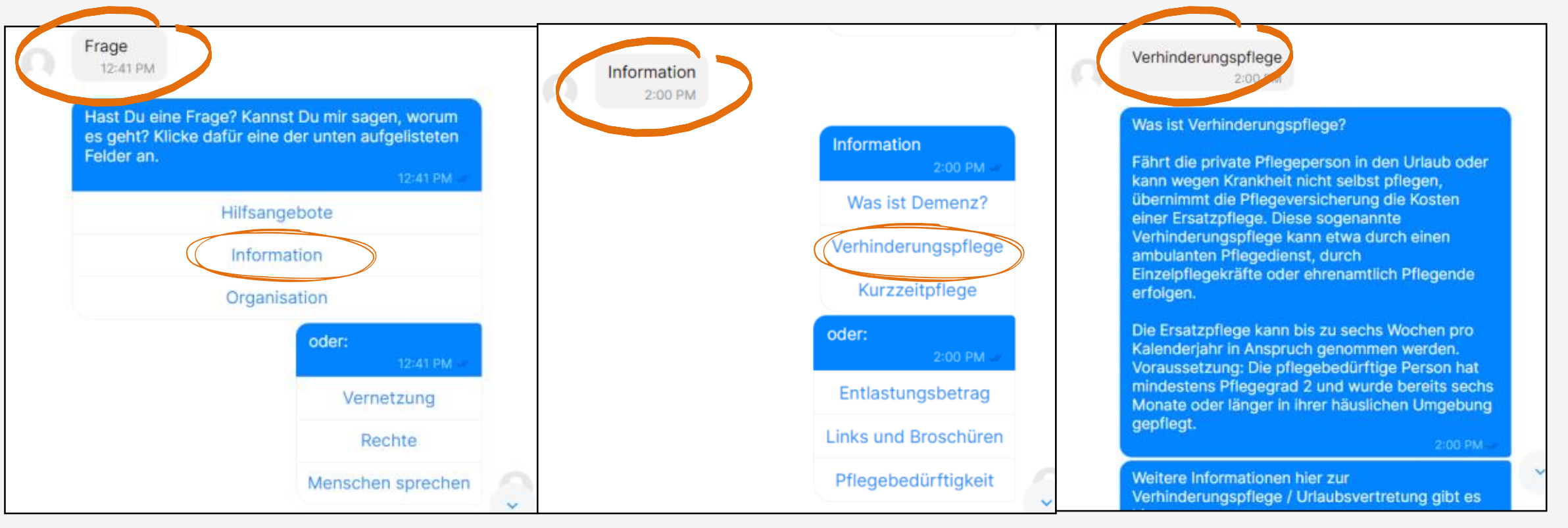

“Ask” free-text & FAQ functions

(1) Care information (What is dementia/ respite care/ short-term care/ the relief amount/ need for care? Helpful links and brochures)

(2) Care organization (nursing levels, applying for a nursing level, medical service/ assessment, long-term care insurance benefits, social assistance, types of care, nursing services & nursing homes)

(3) Support services (Where can I find advice on care/ mental support?)

(4) Networking (Where can I find support/ self-help groups?)

(5) Rights of caregivers (rights in general, rights of workers)

(6) Personal contact with the research team

Sources

1. UCLA Health. *Caregiver training videos*.
   https://www.uclahealth.org/medical-services/geriatrics/dementia/caregiver-education/caregiver-training-videos
   Accessed 2026-03-13.
2. AOK. *PfiFf caregiver training videos*.
   https://aok-pfiff.de/pflegefilme
   Accessed 2026-03-13.
3. Techniker Krankenkasse. *Online yoga video instructions*.
   https://www.tk.de/techniker/aktionen/yoga-online-videoanleitungen-2075370
   Accessed 2026-03-13.
4. Sozialverband VdK Deutschland. *Caregiver rights video materials*.
   https://www.vdk.de/mediathek/
   Accessed 2026-03-13.
