# Supplementary material for: A Co-Designed and Bilingual WhatsApp-Based Chatbot Intervention (PflegeBot) for Informal Caregivers in Germany: Single-Arm Nonrandomized Feasibility Study
Source: JMIR Form Res. 2026 Nov 11;10:e95268. doi: 10.2196/95268 (PMC13754261; doi:10.2196/95268)
Supplement: Supplementary file 2 [file formative-v10-e95268-s002.pdf]

Methods and Supplementary Findings for the Participatory Consultation Workshops

Workshop Procedures and Matrix-Based Descriptive Synthesis

Toward the end of the feasibility phase, once most participants had completed the 12-week intervention cycle, three supplementary consultation workshops were conducted to inform intervention refinement and future implementation. These workshops were formative consultation activities and were not port of the formal feasibility, usability, or acceptability outcome evaluation.

The workshops followed a structured review of the PflegeBot's modules and features and addressed access to the intervention, its implementation, content, usability, and potential future implementation; there was no formal discussion guide. The workshops were attended by both participants with direct experience using the PflegeBot and individuals who had not yet used the intervention. The contributions were therefore treated, depending on the case, either as reported user experiences or as more general evaluations of the intervention concept (see Table A5.1).

The workshop records were summarized using a matrix-based descriptive synthesis (Table A5.2). During review of the records, six descriptive domains were developed: access and onboarding; message delivery and engagement; relevance and personalization; content format, accessibility, and navigation; human support and interactive functions; and implementation and sustainability. Recorded observations were assigned to these domains, and similar, divergent, and complementary observations were compared across workshops. During the synthesis, a distinction was made between contributions from participating informal caregivers, external stakeholders, and members of the research team.

Since the available workshop transcripts were not verbatim records, statements were paraphrased, direct quotes were not included, and neither participant-level frequencies nor formal comparisons between the workshops were calculated.

Table A5.1. Characteristics and documentation of the three consultation workshops

Characteristic	German-language participant workshop	Turkish-language participant workshop	Stakeholder and project-team workshop
**Date and duration**	February 22, 2024; 11:00 AM–1:00 PM	February 23, 2024; 11:00 AM–1:00 PM	February 19, 2024; 12:00 PM–2:00 PM
**Setting**	Gesundheitswerkstatt Osterholz, Bremen	ZIS Gröpelingen, Bremen	BIPS, Bremen
**Language**	German	Turkish	German
**Participating informal caregivers**	4	7 women	Not applicable
**Participants with PflegeBot experience**	3	4	Not applicable
**External stakeholders**	0	0	2, representing DIKS and the Bremen Adult Education Center
**Research-team involvement**	HG moderated; additional project-team members attended	HG moderated; EM attended	HG and TB co-moderated; six research-team members attended in total
**Documentation**	Audio-recorded; detailed non-verbatim record prepared by TF and checked by HG against the recording	Audio-recorded; detailed record and German translation prepared by HG	Contemporaneous notes prepared by TF and HG; no audio recording
**Additional quality check**	Checked by a second researcher against the recording	No second Turkish-language review	Notes prepared by two researchers
**Use of quotations**	None	None	None

Table A5.2. Descriptive synthesis of workshop findings by domain

Domain	German-language participant workshop	Turkish-language participant workshop	Stakeholder and project-team workshop
**Access and onboarding**	Personal assistance with setup and clearer guidance on using WhatsApp on tablets or computers were considered useful. Limited digital confidence and concerns about WhatsApp could restrict access.	Baseline survey, missing login information, and limited experience with digital services were discussed as potential barriers. Personal assistance and digital-skills support were considered important.	The discussion addressed technical requirements and the challenges associated with using an existing commercial messaging platform.
**Message delivery and engagement**	Regular messages were valued as reminders, but some participants described initial pressure to view all materials. Flexible timing and different schedules for weekdays and weekends were discussed.	Daily messages and delivery at 8 PM were often considered difficult to integrate into everyday life. Selectable timing and a lower but still regular frequency were suggested.	Message quantity and timing were discussed as factors affecting sustained engagement.
**Relevance and personalization**	Participants discussed topic selection and personalization but also noted that broad content could introduce users to information that became relevant later.	Tailoring by caregiving relationship, care recipient's condition, and thematic interest were emphasized.	Personalization was considered desirable but potentially resource-intensive and technically complex.
**Content format, accessibility, and navigation**	Shorter materials, clear topic labels, video-length information, and archive, search, or favorites functions were suggested. Some longer CBT-informed materials were considered difficult to integrate into demanding caregiving routines.	Participants favored short, simple information in Turkish, concise introductions, a combination of text and video, and synchronized rather than subtitled videos.	The need for continuing professional review of external content was discussed. One external video was considered inconsistent with the usual professional advice of one stakeholder.
**Human support and interactive functions**	The question function was not sufficiently visible, and repeated reminders were suggested.	Participants discussed the value of a visible live-chat function, personal contact, and links between the chatbot and face-to-face support.	Responsive human support was recognized as requiring defined staff responsibilities and sufficient capacity.
**Implementation and sustainability**	Caregiver groups, care services, and local organizations were discussed as possible dissemination channels.	Local services, community organizations, and caregiver groups were considered important for recruitment, setup, and continued support.	Discussion focused on data protection, dependence on Meta, technical maintenance, staffing, financing, and institutional responsibility for longer-term delivery.
