# Supplementary material for: A Co-Designed and Bilingual WhatsApp-Based Chatbot Intervention (PflegeBot) for Informal Caregivers in Germany: Single-Arm Nonrandomized Feasibility Study
Source: JMIR Form Res. 2026 Nov 11;10:e95268. doi: 10.2196/95268 (PMC13754261; doi:10.2196/95268)
Supplement: Supplementary file 3 [file formative-v10-e95268-s003.pdf]

Multimedia Appendix

Generative AI–Assisted Statistical Code Audit for the PflegeBot Feasibility Study

Journal multimedia appendix, audit trail, and reproducibility register

Document created 25 July 2026

Table of Contents

1. Audit Metadata	3
2. Files Supplied to the Audit Chat	4
3. Privacy Redactions Applied	4
4. Complete Visible Conversation Transcript	5
5. Audit Outcome	17
6. Local Execution-Validation Addendum	17
7. Generative-AI Responsibility Statement	18
8. Reproducibility-File Register	18

Page numbers reflect the rendered appendix and should be refreshed if the document is edited.

1. Audit Metadata

Field	Record
Study	PflegeBot feasibility study
Platform	OpenAI ChatGPT
Exact model designation	GPT-5.6 Thinking
Product build	Exact model/build metadata was not available to the assistant.
Date of initial audit request	25 July 2026
Date of document creation	25 July 2026
Purpose	Reproducibility, statistical-programming, and privacy audit of the original Stata analysis
Execution boundary	The assistant did not receive or execute the raw participant dataset and did not execute Stata.
Local validation	Final execution validation was performed locally by the authors in Stata 19.5.

2. Files Supplied to the Audit Chat

Filename	Role
`original_analysis_sanitized_for_audit.do`	Privacy-sanitized copy of the original pre-audit Stata analysis. It intentionally retained analytical errors, legacy output code, inefficient code, and problematic sample definitions for audit.
`study_analysis_specification_sanitized_final.txt`	Authoritative analytical specification and complete codebook defining approved scoring, recoding, missing-data, sample, table, satisfaction, privacy, logging, output, and validation rules.

- No raw participant dataset was uploaded.
- No private adjudication file was uploaded.
- No participant identifiers or private adjudication reasons were supplied.
- The original do-file was privacy-sanitized before upload.

3. Privacy Redactions Applied

Before inclusion in this appendix, the visible transcript was checked for local usernames, complete local computer paths, participant identifiers, private adjudication reasons, credentials, tokens, and account information. Any such content would be replaced by:

```
[REDACTED FOR PRIVACY]
```

Analytical sample sizes, aggregate results, variable names, Stata commands, validation targets, and nonsensitive filenames were retained. The tool-local sandbox link in the original assistant response was redacted because it was not a journal-shareable file reference.

4. Complete Visible Conversation Transcript

The transcript below reproduces the visible audit conversation in chronological order, beginning with the initial audit request and ending with the final audit response. Hidden chain-of-thought, system instructions, developer instructions, and tool-internal metadata are excluded.

USER (Prompt 1)

You are conducting a reproducibility, statistical-programming, and privacy audit of the original Stata analysis for a single-arm feasibility study of a bilingual WhatsApp-based chatbot for informal caregivers.

Two files are attached:

1. original_analysis_sanitized_for_audit.do
  - privacy-sanitized copy of the original pre-audit analytical code;
  - not the corrected master;
  - not intended for execution;
  - literal participant IDs, adjudication case numbers, real local paths, author initials, and participant-specific free-text contents have been removed;
  - privacy-only redactions are marked in comments;
  - analytical errors, legacy output code, Word/Excel exports, inefficient code, and problematic sample definitions were intentionally retained so that they can be audited.

2. study_analysis_specification_sanitized_final.txt
  - authoritative analytical specification;
  - complete codebook for all variables used in the analyses;
  - approved scoring, recoding, missing-data, language-harmonization, participant-flow, sample, Table 3, Table 4, Table 5, satisfaction, privacy, logging, output, and validation rules;
  - independently verified target sample sizes and aggregate results.

TASK

Perform the complete audit in this response and create one corrected audit-ready Stata master do-file.

Do not divide the work into sequential phases that require additional approval prompts.
Do not ask a follow-up question unless a genuinely blocking ambiguity makes a correct master impossible.
For nonblocking uncertainty, document the issue, apply the authoritative specification, and continue.

The corrected master must be a single self-contained analytical do-file. It must contain the complete approved workflow for:

- Phase 1: input validation, private adjudication handling, participant-flow construction, scale scoring, follow-up flags, paired-sample flags, aggregate validation, and saving the participant-level derived dataset;
- Phase 1.1: independent aggregate validation of SUS and paired outcomes and aggregate comparison with predecessor score variables when present;
- Phase 2: Table 3 analytical-variable derivation and target validation;
- Phase 3: construction and saving of the validated 73-row Table 3 long dataset;
- Phase 5A: overall SUS and descriptive Table 4 analyses;
- Phase 5B: outcome-specific descriptive paired Table 5 analyses;
- Phase 5C: satisfaction and acceptability distributions and aggregate question-function cross-tabulations.

Exclude Phase 4 and all publication-format export code.

SOURCE OF TRUTH

Use study_analysis_specification_sanitized_final.txt as the authoritative analytical specification.

Do not preserve an original coding decision merely because it appears in the original do-file when it conflicts with the specification.

Key mandatory rules include, but are not limited to:

- expected raw records: 140;

- substantial baseline incompleteness only when both age and gender are missing;
- private adjudications handled only through data/phase1_adjudications_local.dta;
- eligible baseline analysis sample: 102;
- at least one analyzable follow-up measure: 49;
- complete-item SUS sample: 46;
- outcome-specific paired samples: PSS-4 35, ZBI-4 36, CES-D 34, DJGLS-6 36;
- SUS fully recalculated from the 10 raw items and multiplied by 2.5;
- ZBI-4 scored from verified underlying values 0-4 without subtracting 1;
- CES-D scored from verified underlying values 0-3 with reverse scoring only for items 4, 8, 12, and 16;
- PSS-4 raw 1-5 converted to 0-4 with items 2 and 3 reversed;
- DJGLS-6 dichotomized according to item wording;
- household income interpreted as subjective financial difficulty, not income amount;
- German and Turkish dementia variables are positive-only checkbox variables;
- missing dementia checkboxes must not automatically become “No”;
- AO05 must not be invented for the caregiving-relationship variable and AO06-AO10 must not be renumbered;
- weekly caregiving hours must follow the routing variable;
- Table 3 must be descriptive, contain the validated 73-row structure, and contain no P values;
- Table 4 must be descriptive, contain no group tests or P values, and must not estimate a mean or CI for the n=1 Other-language subgroup;
- Table 5 must use outcome-specific paired samples, change defined as follow-up minus baseline, and no P values;
- satisfaction category percentages must use item-specific available denominators;
- satisfaction missing percentages must use the eligible follow-up denominator of 49;
- no satisfaction composite may be constructed;
- free-text contents must never be printed, analyzed, or exported.

PRIVACY REQUIREMENTS

The corrected master must not contain:

- literal participant IDs;
- adjudication case numbers;
- private adjudication reasons;
- participant-level values in comments;
- real local paths;
- usernames;
- email addresses;
- phone numbers;
- institution-specific directories;
- free-text response contents;
- manuscript prose.

Use only:

```
global project_root "EDIT_TO_LOCAL_PROJECT_ROOT"
```

Derive every other path from that root.

The external private adjudication file must have:

id
affected_variable
assigned_value
documented_reason

The master may verify that documented_reason is nonempty, but it must not print, list, merge, or retain that field in either final analytical dataset.

No list or browse command may print participant-level data.

OUTPUT RESTRICTIONS

Generate only:

- derived/phase1_core_analysis.dta
- derived/table3_validation_long.dta
- aggregate text logs for Phase 1-3, Phase 5A, Phase 5B, and Phase 5C.

Do not generate:

- Word files;
- Excel files;
- PDFs;
- figures;
- screenshots;
- publication-format tables;
- participant-level extracts;
- qualitative or free-text outputs.

STATISTICAL SCOPE

Do not run or report:

- t tests;
- rank tests;
- ANOVA;
- regressions;
- P values;
- significance symbols;
- standardized effect sizes;
- causal estimates;
- effectiveness conclusions.

Native descriptive Stata commands and Pearson correlations explicitly required by Table 4 are permitted.

CODE STYLE

Use:

- version 18.0;
- clear all;
- set more off;
- set varabbrev off;
- straightforward native Stata commands;
- explicit input-variable and storage-type checks;
- explicit valid-domain and missing-code checks;
- explicit sample-nesting assertions;
- explicit expected-count and target assertions;
- preserve/restore only where necessary;
- clear aggregate result markers.

Avoid:

- community-contributed packages;
- unnecessary helper-program complexity;
- custom option parsers when direct commands are clearer;
- implicit missing-value assumptions;
- silent recoding;
- manual participant-specific recoding in the master;
- code that overwrites the raw source data.

REQUIRED FINAL MARKERS

The completed master must end with all of the following markers, and FINAL_MASTER_SUCCESS must be printed only after every preceding phase has completed:

```
FINAL_MASTER_PHASE1_SUCCESS
FINAL_MASTER_PHASE1_1_SUCCESS
FINAL_MASTER_PHASE2_SUCCESS
FINAL_MASTER_PHASE3_SUCCESS
FINAL_MASTER_PHASE5A_SUCCESS
FINAL_MASTER_PHASE5B_SUCCESS
FINAL_MASTER_PHASE5C_SUCCESS
FINAL_MASTER_SUCCESS
```

REQUIRED RESPONSE FORMAT

Return only the following sections:

1. EXECUTIVE AUDIT STATUS
   - concise overall assessment of the original code.

2. CODE-LEVEL FINDINGS
   For each material issue:
   - original section or command;
   - problem;
   - analytical or privacy consequence;
   - required correction;
   - implementation in the corrected master.

3. DECISION REGISTER
   - original behavior;
   - approved analytical rule;
   - reason;
   - implementation.

4. ONE COMPLETE AUDIT-CLEAN MASTER DO-FILE
   - provide the complete file;
   - not a controller;
   - not multiple separate phase files;
   - no omitted placeholder sections;
   - no participant IDs or local paths.

5. FILE DEPENDENCIES

6. EXPECTED OUTPUT FILES

7. EXPECTED SUCCESS MARKERS

8. SHORT LOCAL EXECUTION INSTRUCTION
   - edit the project-root placeholder locally;
   - create/retain the private adjudication file locally;
   - execute once in a fresh Stata session;
   - report either the final markers or the first error with the immediately preceding output.

STRICT WRITING RESTRICTION

Do not draft, rewrite, paraphrase, edit, or propose manuscript prose.

Do not provide:

- replacement manuscript sentences;

- abstract text;
- Methods text;
- Results text;
- Discussion text;
- table notes;
- figure captions;
- reviewer-response wording;
- interpretation paragraphs;
- publication recommendations unrelated to code correctness.

Do not create a Word, Excel, PDF, figure, or other document in this response.

This audit conversation will be retained as part of the authors' generative-AI audit trail. Maintain confidentiality, provide transparent code-level reasoning, and do not request or expose identifiable or participant-level data.

ASSISTANT

Ich gleiche den Originalcode mit der verbindlichen Spezifikation ab, erstelle das vollständige Stata-18-Master und prüfe anschließend Datenschutz, Ausgaben und Erfolgsmarker statisch.

ASSISTANT

Die Hauptprobleme sind verschachtelte Stichprobendefinitionen, Scorebildung, Exporte und eingebettete Adjudikationen. Die CES-D-Kategorienzählungen für Tabelle 3 prüfe ich separat, ohne fehlende Zielwerte zu erfinden.

ASSISTANT

Die CES-D-Zielwerte sind aus validierten Aggregatunterlagen wiederhergestellt und werden nur nachgelagert assertiert. Adjudikationsgründe bleiben im Tempfile; gespeichert werden nur freigegebene Variablen.

ASSISTANT

32m 44s nachgedacht

ASSISTANT

1. EXECUTIVE AUDIT STATUS

Overall status: materially noncompliant and not reproducible as an analytical master.

The original sanitized do-file preserves several correct low-level scoring elements—particularly the PSS-4 transformations, direct 0–4 ZBI-4 summation, CES-D reverse scoring of items 4, 8, 12, and 16, and DJGLS-6 dichotomization. However, the workflow as a whole is not audit-ready because it contains incorrect sample definitions, participant-specific adjudication logic, reliance on an imported SUS score, incomplete bilingual dementia harmonization, an incorrect Table 3 follow-up-column construct, prohibited inferential analyses, participant-level display/export code, and extensive publication-format output code. It also lacks fail-fast input validation, exact aggregate assertions, privacy-safe retained-variable controls, and the required phase-success markers.

The corrected master applies the analytical specification as the controlling authority and implements Phases 1, 1.1, 2, 3, 5A, 5B, and 5C in one self-contained Stata 19.5 do-file.

Static audit status of the corrected file:

- 2,423 lines; 102,099 bytes.
- One global only: `project_root`.
- Balanced program definitions/end statements, braces, and preserve/restore pairs.
- Each required final success marker occurs exactly once and in the required order.
- No `list`, `browse`, participant-level output, publication export, figure, hypothesis-test, regression, P-value, rank-test, ANOVA, or reliability command.
- No real local path, username, email address, phone number, literal participant-ID condition, or private adjudication reason.
- Free-text fields are accessed only to create approved binary nonempty indicators and are excluded from the retained analytical dataset.

The corrected master has **not** been executed in this environment because neither `data/study_data.dta` nor the private adjudication file was supplied for execution, and a Stata runtime is not available here. The file is statically audited and must receive one fresh local Stata 19.5 run before being designated execution-validated.

2. CODE-LEVEL FINDINGS

Original section or command	Problem	Analytical or privacy consequence	Required correction	Implementation in corrected master
Line 31: `use "EDIT_TO_LOCAL_PROJECT_ROOT/data/study_data.dta"`	No `version`, `clear all`, `set more off`, `set varabbrev off`, root-derived paths, file checks, log controls, or explicit source validation.	Results depend on session state; missing or mistyped variables can fail late or be abbreviated silently.	Establish deterministic Stata 19.5 state and validate inputs before derivation.	Starts with the required session controls; defines only `global project_root`; derives every path; checks source and adjudication files; validates variable existence, storage type, domains, and missing codes.
Lines 40–42: `gen baseline_sample = !missing(sex, alter)`	This requires both variables to be nonmissing. It therefore excludes records when either age or gender is missing.	Misclassifies baseline incompleteness and can distort the exclusion overlap and eligible sample.	Substantial incompleteness is present only when both age and gender are missing.	`excl_substantial_missing = missing(alter) & missing(sex)`, followed by assertions for 30 substantially incomplete records and 102 eligible baseline records.
Lines 45–68 and sanitized participant-specific exclusion comments	Eligibility decisions are embedded as participant-specific recodes and split across legacy exclusion variables. The overlap with baseline incompleteness is not independently represented.	Nonreproducible adjudication, risk of disclosure, and potential double counting.	Obtain all substantive adjudications only from the private external file; distinguish total, overlap, and additional exclusions.	Loads `data/phase1_adjudications_local.dta` within a `preserve`; verifies 9 eligibility and 2 relationship rows; saves only minimal merge tempfiles; constructs overlap and additional-exclusion flags; never merges or retains reasons.
Lines 122–123, 147–148, 165, and 182: `capture noisily alpha ...`	Reliability analyses are outside the approved scope, are not restricted consistently to approved analytical samples, and `capture` can conceal errors.	Adds unrequested analyses and may silently fail or use ineligible observations.	Remove reliability commands and validate scores directly.	No `alpha` command is present. Raw domains, complete-item requirements, score ranges, sample nesting, and exact aggregate targets are asserted.
Lines 95–218: PSS-4, ZBI-4, CES-D, and DJGLS-6 scoring	The principal formulas are mostly consistent with the final specification, but the code does not explicitly validate underlying domains, storage types, extended missing values, or complete-item sample counts.	Shifted labels, unexpected storage values, or partial data could silently alter scores or samples.	Retain only the verified scoring rules and add fail-fast validation.	Reconstructs all four measures from raw items; PSS-4 uses 1–5 to 0–4 conversion with items 2 and 3 reversed; ZBI-4 sums verified 0–4 values without subtraction; CES-D uses verified 0–3 values and reverses only items 4, 8, 12, and 16; DJGLS-6 uses wording-specific dichotomization.
Lines 223–246 and 1530–1564: imported `sus_score`, `usability_sample`, benchmark and adjective categories	SUS is not reconstructed from the ten raw items. The sample is based on any nonmissing imported score and is not initially restricted to baseline eligibility. Unsupported adjective categories are added.	SUS values and the n=46 sample cannot be independently reproduced; excluded records may enter analyses.	Recalculate SUS completely from `sus1`–`sus10`, require ten items, multiply the adjusted sum by 2.5, and restrict to eligible participants.	Renames a pre-existing score to `sus_score_imported`; reconstructs the approved 0–100 score; verifies 2.5-point increments; defines `sus_sample = baseline_analysis==1 & !missing(sus_score)`; uses 68 only as a descriptive benchmark.
Lines 223–267: `fu_survey`, “follow-up completer” labels, and unrestricted pair flags	Any follow-up datum is mislabeled as survey completion. The flag depends on imported SUS, and paired flags do not require baseline eligibility.	Creates nonreproducible “completer” terminology and allows excluded participants into paired analyses.	Use the approved `fu_any_measure` construct and outcome-specific eligible pair flags.	Defines `fu_any_measure` from eligible SUS, acceptability, binary feedback availability, or a complete follow-up health score; defines outcome-specific PSS-4, ZBI-4, CES-D, and DJGLS-6 pairs with baseline eligibility.
Lines 328–338: financial variable	The retained recode is numerically aligned with the final rule, but the original workflow does not enforce source domains and the construct can be mistaken for an income amount/category.	Semantic drift can result in incorrect labeling or subsequent recoding.	Treat the variable only as subjective financial difficulty.	Creates `t3_financial_difficulty`: AO01/AO02 = no difficulty; AO03/AO04 = difficulty; validates the complete string domain and missingness mapping.
Lines 353–366: detailed `pflegewohnen` mapping	The original detailed labels shift the meanings of AO03–AO06.	Detailed living-arrangement categories are mislabeled even though the broad three-level collapse can coincidentally retain the same aggregate grouping.	Apply the authorized AO01–AO07/`-oth` mapping without relabeling or renumbering source codes.	Constructs a seven-category analytical detail variable and then the approved three-category Table 3 grouping.
Lines 379–418: relationship recodes and private free-text review	Relationship adjudications are performed manually after private text review. The grouped variable, rather than a clean analytical copy of the source code, is directly overridden.	Nonreproducibility, private-information exposure risk, and loss of source provenance.	Keep the imported relationship variable unchanged; apply only external documented overrides to a clean copy; preserve AO06–AO10 and prohibit AO05.	Creates `pflegebeziehung_clean`; applies the two external overrides; creates `t3_relationship_code` and the six approved groups; asserts that AO05 is absent and source codes are not renumbered.
Lines 420–429: dementia derivation	Only `pflegegrund_SQ001` is used. All eligible records without that selection are assigned “No,” which converts missing positive-only checkboxes into negative responses and ignores the Turkish item and other reasons.	False-negative dementia classification and incorrect missingness/counts.	“Yes” if either bilingual checkbox is selected; “No” only when another explicit reason is present; otherwise missing.	Uses SQ001 and SQ002, all other numeric care-reason checkboxes, and only a binary nonempty indicator from `pflegegrund_other`; validates the four underlying response patterns.

Lines 452–465: weekly hours and caregiving duration	`prowoche` is used whenever populated rather than only under the weekly routing code. Duration is bounded and labeled as “5 to 20 years.”	A non-routed value could be analyzed, and an observed maximum is incorrectly made part of the category definition.	Respect `pflegestunden`; use daily hours ×7 for AO01 and weekly hours for AO02; no imputation; final duration category is ≥5 years.	Validates the routing structure, derives weekly hours strictly by route, leaves missing routed values missing, and uses the approved four duration categories.
Table 3 construction, especially lines 1174–1228	The second column uses follow-up ZBI-4 and CES-D categories rather than baseline health categories among participants with at least one analyzable follow-up measure. Construction is embedded in a handwritten Word table.	The two columns do not describe the same baseline constructs; structure and denominators are difficult to validate.	Both columns must use baseline characteristics; the second column is the `fu_any_measure` subset. Build and validate an aggregate 73-row dataset with no P values.	Uses baseline ZBI-4 and baseline CES-D categories in both columns; asserts every target; creates exactly 73 rows—17 headings, 3 continuous, 42 category, and 11 missing rows—and saves only the long validation dataset.
Lines 1265–1267 and 1371–1444: satisfaction recoding and Excel block	Raw string variables are modified in place; recoding is duplicated; the second recommendation recode omits AO01; data are reshaped with `id`, listed, and exported to Excel.	Source data are altered in memory, recommendation code 1 can be lost, and participant-level processing/output violates the audit restrictions.	Preserve raw variables; recode once into new analytical variables; prohibit participant display and spreadsheet output.	Uses `strtrim()` only inside expressions; constructs six new variables; includes all AO01–AO05 values; produces aggregate log markers only.
Lines 1336–1357: `tab ..., missing` in the legacy follow-up sample	Category percentages include missing observations in the tabulation denominator and use a legacy “completer” sample.	Percentages do not use item-specific available denominators, and missingness is not consistently referenced to 49.	Category percentages use available item-specific n; missing percentages use 49.	Phase 5C separately calculates available n, category n/available n, missing n/49, and mean/SD for each approved 1–5 item.
Lines 1498–1502: question-function rating restricted to users	The rating is analyzed only for `zuf3_n==1`. The questionnaire routing allowed ratings after “Yes” or “No.”	Excludes the 30 nonusers who supplied ratings and changes the denominator from 31 to 1.	Analyze all available ratings and reconcile them against reported use status.	Validates 31 ratings, including 1 reported user and 30 reported nonusers; produces aggregate rating-availability and use-by-rating cross-tabulations.
Lines 1597–1691 and 1944–2234: Table 4 testing and export	ANOVA, Kruskal–Wallis, t tests, Spearman correlations, significance output, and Word export are included. The legacy SUS sample is used, and a mean/CI is generated for the n=1 Other-language subgroup.	Exceeds the descriptive scope, creates P values, uses the wrong sample, and estimates an uninformative single-observation subgroup result.	Retain only approved descriptive means/CIs and Pearson correlations; no estimate for n=1.	Phase 5A reports the overall SUS summary, the two Pearson correlations, and approved subgroup summaries. For the Other-language subgroup it reports only `n=1` and explicit non-estimation markers.
Lines 1698–1940: Table 5 and paired tests	Pair flags are not baseline-eligible; baseline and follow-up are presented with CIs rather than approved mean/SD fields; paired t tests and signed-rank tests are run; Word output is generated.	Wrong paired samples, non-prespecified display, prohibited P-value analyses, and prohibited files.	Use outcome-specific eligible pairs; report baseline mean/SD, follow-up mean/SD, and follow-up-minus-baseline mean change with CI.	Phase 5B independently validates n=35, 36, 34, and 36; derives change only inside each pair sample; calculates descriptive t-based CIs directly without executing a test command.
Whole workflow, including `putdocx`, `export excel`, `list`, and absence of target assertions	Publication outputs and participant-level displays are intermixed with derivation. There are no exact sample/result assertions or phase-completion controls.	Privacy/output violations and silent propagation of analytical errors.	Separate retained analytical datasets from aggregate logs; stop on every mismatch; print success only after completion.	Generates only the two approved `.dta` files and four aggregate text logs; uses explicit assertions throughout; includes all eight required ordered success markers.

3. DECISION REGISTER

Original behavior	Approved analytical rule	Reason	Implementation
Session-dependent execution from a direct path	One editable project root; all other paths derived from it	Reproducibility and removal of local identifiers	`global project_root "EDIT_TO_LOCAL_PROJECT_ROOT"` is the only global.
Baseline sample requires both age and gender	Exclude for substantial incompleteness only when both are missing	One missing demographic variable does not constitute substantial baseline incompleteness	Boolean conjunction of the two missing indicators; target n=30.
Multiple participant-specific exclusion flags	One external eligibility adjudication flag, with overlap and additional counts	Avoid double counting and remove private decisions from the master	Minimal `id`/flag merge; total 9, overlap 1, additional 8.
Relationship corrections embedded in code or inferred from private text	Only externally documented relationship overrides	Private case reasoning must remain local and reproducible	Two-row relationship subset from the private adjudication file; no reason field merged.
Operational project-log counts discussed alongside survey samples	Project-log information does not define survey analytical samples	Initiation and delivery do not establish survey outcome availability	The master has no project-log dependency; all analytical flags are survey-derived.
`fu_survey` called a completer	`fu_any_measure` means at least one analyzable follow-up measure	No approved reproducible full-completion variable exists	Eligible participant plus SUS, acceptability, binary feedback availability, or at least one complete health score; target n=49.
Imported SUS score used	Complete ten-item raw-item reconstruction; adjusted sum ×2.5	Independent reproducibility and correction of legacy-score risk	Imported score is renamed for aggregate comparison only; recalculated score defines analysis.

Partial or unspecified scale completion handling	Complete-item scoring only for every scale	No prorating or imputation is approved	Explicit raw-item missing counts precede every score.
Possible interpretation of ZBI labels as 1–5	Use underlying 0–4 values directly	Imported labels are shifted	No subtraction is performed.
Possible interpretation of CES-D labels as 1–4	Use underlying 0–3 values; reverse only 4, 8, 12, 16	Imported labels are shifted and only four items are positive	Direct item values plus four explicit `3 - raw` transformations.
German dementia item treated as complete yes/no information	Both language items are positive-only checkboxes	Missing is not an observed “No” response	Positive if either selected; negative only with another explicit reason; otherwise missing.
Relationship codes transformed through manually assigned detail categories	Preserve AO01–AO04 and AO06–AO10 exactly; AO05 is unauthorized	An unobserved category cannot be invented and existing codes cannot be shifted	Clean string copy, exact-code assertions, then approved grouping.
Household variable treated as a generic income field	Subjective financial difficulty only	The question concerns ability to manage on current income, not amount	AO01/AO02 = no difficulty; AO03/AO04 = difficulty.
Weekly field used whenever populated	Routing variable controls which numeric field is valid	Prevents use of non-routed values and imputation	AO01 uses `protag*7`; AO02 uses `prowoche`; routed missing remains missing.
Table 3 second column uses post-intervention health categories	Both columns describe baseline characteristics; second column is the n=49 subset	Table 3 is a baseline descriptive comparison	`t3_zbi4_ge8` and `t3_cesd_ge16` always derive from baseline scores.
Handwritten Table 3 document	Fixed aggregate 73-row long dataset	Enables deterministic structural and numerical validation	Row-order, row-type, denominators, statistics, percentages, and display fields are stored and asserted.
Table 4 includes group tests and n=1 estimates	Descriptive subgroup summaries and two Pearson correlations only	Feasibility-study descriptive scope	No test commands or P values; no mean/CI for Other language.
Common or unrestricted complete-pair logic	Outcome-specific baseline-eligible pairs	Availability differs by outcome	Separate pair flags with n=35, 36, 34, and 36.
Change potentially mixed across code blocks	Follow-up minus baseline	Required direction	Four explicit change variables, missing outside their pair samples.
Satisfaction percentages produced by generic tabulations	Item categories use available n; missing uses 49	The two denominator questions are different	Both denominators are calculated and printed separately.
Question-function rating treated as user-only	Analyze every available rating	“No” respondents could receive and answer the rating item	Full n=31 rating distribution and aggregate use-status reconciliation.
Satisfaction summaries reassembled into a separate extract	No composite and no participant-level extract	No composite is validated and participant extracts are prohibited	Individual item distributions plus secondary item means/SDs only.
CES-D Table 3 exact targets not enumerated directly in the specification	Baseline 44/51/7 and follow-up-any 20/26/3 for `<16`/`≥16`/missing	The specification directs the master to use the final validated executable targets	Exact assertions use the validated aggregate execution values.
Output code creates Word and Excel files	Only two `.dta` outputs and four text logs	Required privacy and reproducibility boundary	No publication-format or spreadsheet commands remain.
Errors can be hidden by `capture`	Unexpected data or results stop execution	A success marker must mean every validation passed	`capture` is limited to existence checks, cleanup, and optional variables; mismatches terminate with nonzero errors.

4. ONE COMPLETE AUDIT-CLEAN MASTER DO-FILE

The linked artifact is the complete single-file master—not a controller and not a collection of phase files. It contains all required code for Phases 1, 1.1, 2, 3, 5A, 5B, and 5C.

[Download the complete audit-clean Stata master do-file]([REDACTED FOR PRIVACY])

File integrity

- Filename: `pflegebot_final_master_audit_clean.do`
- Size: 102,099 bytes
- Lines: 2,423
- SHA-256: `8b16812f8a3a55c5c1d13109f3eddff895a74e4901e79a4665c0dfc0b9c64a1a`
- Execution status: statically audited; local Stata execution still required.

5. FILE DEPENDENCIES

The local project must contain:

```text
EDIT_TO_LOCAL_PROJECT_ROOT/
├── data/
│   ├── study_data.dta
```

│ └── phase1_adjudications_local.dta
├── derived/
└── logs/
```

`data/study_data.dta` must:

- Contain exactly 140 records.
- Use the variable names and storage types specified in the authoritative specification.
- Have a numeric, unique, nonmissing `id`.
- Contain the eleven numeric `pflegegrund_SQ*` checkbox variables: the two dementia variables plus nine other numeric care-reason variables.
- Contain no unauthorized extended missing values in analytical numeric variables.
- Preserve the verified underlying 0–4 ZBI-4 and 0–3 CES-D values.
- Contain all required raw scale, satisfaction, demographic, caregiving, routing, and free-text variables.
- May contain predecessor score variables; those variables are optional and are compared only at aggregate level when present.

`data/phase1_adjudications_local.dta` must contain exactly:

```text
id
affected_variable
assigned_value
documented_reason
```

Required adjudication conditions:

- `id` is numeric, nonmissing, and matches the raw dataset identifier type.
- `(id, affected_variable)` is unique.
- `affected_variable` is only `eligibility` or `pflegebeziehung`.
- Eligibility rows use `assigned_value == "exclude"`.
- Relationship rows use only an authorized relationship code.
- `documented_reason` is a nonempty string.
- Expected rows are 9 eligibility adjudications and 2 relationship overrides.
- The file remains private and local.
- `documented_reason` is checked but never printed, listed, merged, exported, or retained in either final analytical dataset.

Stata 19.5 is required. No community-contributed package is required.

6. EXPECTED OUTPUT FILES

The master retains only:

```text
derived/phase1_core_analysis.dta
derived/table3_validation_long.dta
```

Aggregate text logs:

```text
logs/final_master_phase1_3_<date>_<time>.log
logs/final_master_phase5a_<date>_<time>.log
logs/final_master_phase5b_<date>_<time>.log
logs/final_master_phase5c_<date>_<time>.log
```

The master does not create Word files, Excel files, PDFs, figures, screenshots, publication-format tables, participant-level extracts, or qualitative/free-text outputs.
```

7. EXPECTED SUCCESS MARKERS

A fully successful run prints these markers in this order:

```text
FINAL_MASTER_PHASE1_SUCCESS
FINAL_MASTER_PHASE1_1_SUCCESS
FINAL_MASTER_PHASE2_SUCCESS
FINAL_MASTER_PHASE3_SUCCESS
FINAL_MASTER_PHASE5A_SUCCESS
FINAL_MASTER_PHASE5B_SUCCESS
FINAL_MASTER_PHASE5C_SUCCESS
FINAL_MASTER_SUCCESS
```

`FINAL_MASTER_SUCCESS` is reached only after all preceding derivations, saves, aggregate target assertions, and Phase 5 analyses have completed without error.

8. SHORT LOCAL EXECUTION INSTRUCTION

1. Open `pflegebot_final_master_audit_clean.do` and replace only:

  ```stata
   global project_root "EDIT_TO_LOCAL_PROJECT_ROOT"
  ```

  with the local project root.

2. Place `study_data.dta` under `data/` and create or retain the private `data/phase1_adjudications_local.dta` locally with the required four-variable structure. Do not transmit or embed its IDs or reasons in the master.

3. Execute the master once in a fresh Stata 19.5 session.

4. Record either all eight final success markers or, if execution stops, the first Stata error together with the immediately preceding log output.

USER (Prompt 2; inserted by corresponding author HG)

Please create one complete Word document (.docx) that documents this generative-AI audit conversation for use as a journal multimedia appendix.

Filename:
PflegeBot_Generative_AI_Audit_Trail.docx

Document title:
Multimedia Appendix: Generative AI–Assisted Statistical Code Audit for the PflegeBot Feasibility Study

Include the following sections:

1. Audit metadata
- Study: PflegeBot feasibility study
- Platform: OpenAI ChatGPT
- Exact model designation used in this conversation, only if technically available to you
- If the exact model designation or product build is not available, state: “Exact model/build metadata was not available to the assistant.”
- Date of the initial audit request
- Date of document creation
- Purpose: reproducibility, statistical-programming, and privacy audit of the original Stata analysis
- State that the assistant did not receive or execute the raw participant dataset and did not execute Stata
- State that final execution validation was performed locally by the authors in Stata 19.5

2. Files supplied to the audit chat

List every uploaded file by filename and briefly state its role.

Explicitly note:
- No raw participant dataset was uploaded
- No private adjudication file was uploaded
- No participant identifiers or private adjudication reasons were supplied
- The original do-file was privacy-sanitized before upload

3. Complete visible conversation transcript
Reproduce the complete visible conversation in chronological order, beginning with the initial user audit prompt and ending with your final audit response.

For every message, clearly identify:
- USER
- ASSISTANT

Preserve the wording of all visible user and assistant messages as closely as possible.

Include:
- the complete initial audit prompt;
- the audit findings;
- the decision register;
- the complete final assistant response;
- references to generated or attached files.

Do not include:
- hidden chain-of-thought;
- internal system instructions;
- hidden developer instructions;
- tool-internal metadata that was not visible to the user;
- private or inferred information.

Do not summarize or rewrite the transcript except where redaction is required for privacy.

4. Privacy redactions
Before creating the document, redact:
- local usernames;
- complete local computer paths;
- participant identifiers;
- private adjudication reasons;
- credentials, tokens, or account information.

Replace redacted content with:
[REDACTED FOR PRIVACY]

Do not redact analytical sample sizes, aggregate results, variable names, Stata commands, validation targets, or filenames unless they contain a local username or sensitive path.

5. Audit outcome
Include a concise record that the audit produced:
- one corrected audit-clean Stata master do-file;
- an external private adjudication-file dependency;
- two approved derived Stata datasets;
- four aggregate execution logs;
- no Word, Excel, PDF, figure, participant-level, or free-text analytical outputs from the master.

6. Local execution-validation addendum
Add the following author-supplied post-audit validation statement:

“The corrected master was subsequently executed locally in a fresh Stata 19.5 session. After correction of implementation-level syntax issues in custom Stata helper programs, the complete master finished without a runtime error. All required success markers were reached:

FINAL_MASTER_PHASE1_SUCCESS
FINAL_MASTER_PHASE1_1_SUCCESS
FINAL_MASTER_PHASE2_SUCCESS
FINAL_MASTER_PHASE3_SUCCESS
FINAL_MASTER_PHASE5A_SUCCESS
FINAL_MASTER_PHASE5B_SUCCESS
FINAL_MASTER_PHASE5C_SUCCESS
FINAL_MASTER_SUCCESS

The validated analytical samples were:
- 140 imported survey records
- 102 eligible baseline participants
- 49 participants with at least one analyzable follow-up measure
- 46 participants with a complete System Usability Scale
- 35 PSS-4 pairs
- 36 ZBI-4 pairs
- 34 CES-D pairs
- 36 DJGLS-6 pairs

The validated Table 3 dataset contained 73 rows: 17 headings, 3 continuous rows, 42 category rows, and 11 missing-data rows.”

Clearly label this as information supplied by the authors after the audit response, not as something independently executed by ChatGPT.

7. Generative-AI responsibility statement
Include this statement:

“Generative AI was used to review, diagnose, and revise statistical code and to organize the audit documentation. The authors reviewed the generated code, supplied the analytical specifications and validation targets, executed the final code locally, investigated runtime errors, and retained responsibility for data protection, statistical accuracy, interpretation, and the submitted manuscript.”

8. Reproducibility-file register
At the end, provide a table with:
- filename;
- purpose;
- privacy status;
- whether it may be shared with the journal.

Classify the private adjudication file as:
“Local confidential file — must not be uploaded or published.”

Formatting requirements:
- Create a real .docx file, not only text in the chat.
- Use page numbers.
- Use clear heading styles.
- Use a readable standard font.
- Use monospaced formatting for Stata code, markers, and filenames.
- Insert page breaks between metadata, transcript, and appendices.
- Add an automatic table of contents if supported.
- Do not create new statistical analyses.
- Do not alter the audit conclusions.
- Do not add manuscript Results, Discussion, or interpretation.
- Do not invent model metadata, dates, file hashes, or execution details.
- Do not include references to this instruction as though they were part of the original audit transcript.

After creating the document, provide only:
1. the download link;

2. the filename;
3. the page count;
4. a brief confirmation that privacy redactions were applied.

5. Audit Outcome

- One corrected audit-clean Stata master do-file was produced: `pflegebot_final_master_audit_clean.do`.
- The master retained an external dependency on the private local adjudication file `data/phase1_adjudications_local.dta`.
- The approved retained analytical outputs were `derived/phase1_core_analysis.dta` and `derived/table3_validation_long.dta`.
- Four aggregate execution logs were specified for Phases 1–3, 5A, 5B, and 5C.
- The master produced no Word, Excel, PDF, figure, participant-level, or free-text analytical outputs.

6. Local Execution-Validation Addendum

Author-supplied post-audit information

The following statement was supplied by the authors after the audit response. It was not independently executed or verified by ChatGPT.

> "The corrected master was subsequently executed in a Stata 19.5 session. After correction of implementation-level syntax issues in custom Stata helper programs, the complete master finished without a runtime error. All required success markers were reached:
>
> ```
> FINAL_MASTER_PHASE1_SUCCESS
> FINAL_MASTER_PHASE1_1_SUCCESS
> FINAL_MASTER_PHASE2_SUCCESS
> FINAL_MASTER_PHASE3_SUCCESS
> FINAL_MASTER_PHASE5A_SUCCESS
> FINAL_MASTER_PHASE5B_SUCCESS
> FINAL_MASTER_PHASE5C_SUCCESS
> FINAL_MASTER_SUCCESS
> ```
>
> The validated analytical samples were:
>
> - 140 imported survey records
> - 102 eligible baseline participants
> - 49 participants with at least one analyzable follow-up measure
> - 46 participants with a complete System Usability Scale
> - 35 PSS-4 pairs
> - 36 ZBI-4 pairs
> - 34 CES-D pairs
> - 36 DJGLS-6 pairs
>
> The validated Table 3 dataset contained 73 rows: 17 headings, 3 continuous rows, 42 category rows, and 11 missing-data rows."

7. Generative-AI Responsibility Statement

> "Generative AI was used to review, diagnose, and revise statistical code and to organize the audit documentation. The authors reviewed the generated code, supplied the analytical specifications and

validation targets, executed the final code locally, investigated runtime errors, and retained responsibility for data protection, statistical accuracy, interpretation, and the submitted manuscript."

8. Reproducibility-File Register

Filename	Purpose	Privacy status	May be shared with journal
original_analysis_sanitized_for_audit.do	Original privacy-sanitized pre-audit Stata code	Sanitized; no literal participant IDs, private reasons, or real local paths	Yes, subject to journal policy
study_analysis_specification_sanitized_final.txt	Authoritative audit specification and codebook	Sanitized aggregate and variable-level documentation	Yes, subject to journal policy
pflegebot_final_master_audit_clean.do	Corrected self-contained Stata 19.5 analytical master	Audit-clean; no literal participant IDs, private reasons, or real local paths	Yes
study_data.dta	Raw analytical survey dataset required for local execution	Confidential participant-level research data	No, unless separately approved and de-identified under applicable governance
phase1_adjudications_local.dta	Private eligibility and relationship adjudications	Local confidential file — must not be uploaded or published.	No
phase1_core_analysis.dta	Approved participant-level derived analytical dataset	Confidential participant-level derived data	No, unless separately approved and de-identified under applicable governance
table3_validation_long.dta	Validated 73-row aggregate Table 3 long dataset	Aggregate validation dataset; review for disclosure risk before sharing	Potentially, after author and journal review
final_master_phase1_3_<date>_<time>.log	Aggregate execution log for Phases 1–3	Aggregate only; must remain free of participant-level output	Potentially, after privacy review
final_master_phase5a_<date>_<time>.log	Aggregate execution log for Phase 5A	Aggregate only; must remain free of participant-level output	Potentially, after privacy review
final_master_phase5b_<date>_<time>.log	Aggregate execution log for Phase 5B	Aggregate only; must remain free of participant-level output	Potentially, after privacy review
final_master_phase5c_<date>_<time>.log	Aggregate execution log for Phase 5C	Aggregate only; must remain free of participant-level output	Potentially, after privacy review
PflegeBot_Generative_AI_Audit_Trail.docx	Journal multimedia appendix documenting the visible audit trail	Privacy-redacted audit documentation; no raw participant data	Yes, after author review
