# Supplementary material for: A Co-Designed and Bilingual WhatsApp-Based Chatbot Intervention (PflegeBot) for Informal Caregivers in Germany: Single-Arm Nonrandomized Feasibility Study
Source: JMIR Form Res. 2026 Nov 11;10:e95268. doi: 10.2196/95268 (PMC13754261; doi:10.2196/95268)
Supplement: Supplementary file 4 [file formative-v10-e95268-s004.pdf]

Methods and Supplementary Findings for Open-Ended Follow-Up Feedback

Data Collection and Descriptive Categorization

In the follow-up survey, participants had the opportunity to provide open-ended feedback on the following question: “Is there anything you noticed while using PflegeBot that you would like to share with us?”. The feedback was collected via the online follow-up survey or through telephone follow-up interviews with participants who had not completed the online survey. The telephone interviews included questions about usability and acceptability; exploratory health outcomes were not assessed.

Feedback was available from 27 participants: 21 submitted written survey responses, five provided feedback during a telephone-administered survey, and one provided both written and telephone feedback. Two responses contained no substantive feedback and were retained for documentation purposes but excluded from the descriptive categorization.

Open-ended feedback was systematically reviewed and grouped into inductively developed descriptive categories. One researcher fluent in German and Turkish reviewed all substantive responses in their original language, identified relevant statements, and developed preliminary category labels and definitions. Turkish-language responses were subsequently translated into German for secondary review. A second researcher reviewed all German-language responses, the German translations of Turkish-language responses, the category assignments, category definitions, and category summaries. The final classifications and descriptions were agreed through discussion; the material was not independently double-coded. The descriptive categories, definitions, main subtopics, participant counts, and illustrative written responses are presented in Table A4.1.

Researcher annotations and interpretations contained in the working material were excluded from the descriptive categorization. Paraphrased telephone field notes contributed to the categorization but were not used as illustrative quotations; verbatim participant statements documented in quotation marks were eligible for quotation. The feedback was not linked to quantitative participant characteristics, and no subgroup comparisons were conducted.

Table A4.1. Descriptive categories and supplementary findings

Descriptive category	Definition	Main subtopics	Participants contributing feedback, n	Illustrative written response
**Relevance and tailoring**	Extent to which the content corresponded to the participant's caregiving situation, current needs, prior knowledge, care recipient's condition, or geographical context	Topic relevance; condition-specific information; prior knowledge; regional relevance; topic selection and personalization	15	"I would like the option to narrow down the topics, for example, to information on long-term care insurance or to relaxation exercises only."
**Delivery intensity and timing**	Experiences concerning the frequency, sequence, timing, and manageability of the delivered messages	Information overload; limited time; processing time; evening delivery; preferred frequency; later retrieval	14	"The information sometimes came too quickly one after another. I needed some time in between to process everything."
**Perceived benefit and acceptability**	Positive or negative assessments of the intervention and reported practical, informational, or emotional value	Overall satisfaction; learning; practical actions; recommendation; relaxation exercises; emotionally burdensome content	10	"I really liked the short and effective exercises and meditations for family caregivers."[a]
**Format, usability, and navigation**	Experiences concerning content presentation, technical use, external links, devices, readability, and retrieval of information	Content length; video format; smartphone versus tablet; visual accessibility; external links; search and navigation	8	"I preferred the self-produced contributions. My overall impression was that the content was often too long to maintain sufficient attention."

[a]Translated from Turkish by HG. All other illustrative responses were translated from German into English. Multiple categories could be assigned to each participant; category counts therefore do not sum to the number of participants providing substantive feedback. Telephone responses documented as paraphrased notes were not used as illustrative responses.
